# A Therapist-Configurable Serious Game System for Upper-Limb Neurorehabilitation: A Pilot Feasibility Study

**DOI:** 10.3390/s26185956

**Published:** 2026-09-20

**Authors:** Juan J. Sánchez-Gil, Aurora Sáez, Juan José Ochoa-Sepúlveda, Rafael López-Luque, Laura Muñoz-Millán, Eduardo Cañete-Carmona

**Affiliations:** 1 Departamento de Ingeniería Electrónica y de Computadores, Universidad de Córdoba, Edificio Leonardo Da Vinci, Campus de Rabanales, 14071 Córdoba, Spain; 2 Instituto de Neurociencias, Hospital Cruz Roja, P.º de la Victoria, 14004 Córdoba, Spain

**Keywords:** upper-limb rehabilitation, gamification, neurorehabilitation, serious games, RGB sensing, pilot feasibility

## Abstract

Upper-limb neurorehabilitation requires adaptable practice. This pilot feasibility study evaluated a therapist-configurable serious-game system in which a handle equipped with an RGB sensor, embedded processing, and wireless communication converted detections of reconfigurable colored targets into control events for 17 games. Thirteen neurological patients began six supervised sessions over three weeks alongside usual care; 11 completed them (84.6%). Including one session completed by each participant who withdrew, 68 sessions were delivered overall (approximately 34 h of scheduled time). From a sensing-operability perspective, no session ended prematurely because of a sensing or other technical failure; RGB recalibration was required in 4 sessions (5.9%). Measures included session logs, System Usability Scale, Intrinsic Motivation Inventory, In-Game Game Experience Questionnaire, Pittsburgh Rehabilitation Participation Scale, Box and Block Test, and exploratory movement analysis. Mean SUS was 76.1 ± 12.6; IMI interest/enjoyment and perceived competence were 6.47/7 and 6.39/7; exploratory post-activity iGEQ positive affect was 3.64/4; and PRPS means ranged from 4.64 to 5.45/6. BBT scores increased descriptively in 11 of 12 assessed affected limbs. The system was feasible and acceptable as a supervised adjunct, but exploratory findings do not establish clinical efficacy.

## 1. Introduction

Neurological conditions such as stroke, traumatic brain injury, brain tumors, and peripheral neurological disorders can cause motor, cognitive, speech, and emotional impairments [1,2], which often require early and ongoing multidisciplinary rehabilitation to reduce long-term disability [3]. Among these conditions, stroke represents one of the most prevalent and clinically relevant causes of acquired disability in the upper extremities [4] and, therefore, has become a key application area for gamified neurorehabilitation technologies. However, the therapeutic requirements addressed by these systems, such as repetitive task-oriented practice, feedback, personalization, and motivation, are also relevant for broader populations of patients undergoing neurological rehabilitation.

Gamification introduces game elements into non-game contexts to improve motivation and adherence to therapy [5]. Beyond this, Serious Games (SGs) provide structured, task-oriented practice, immediate feedback, progress monitoring, and adaptable challenges, making them especially valuable in neurorehabilitation [6]. Their therapeutic effectiveness depends not only on engagement, but also on their alignment with rehabilitation goals and on factors such as intensity, task specificity, safety, and accessibility [6,7].

The system evaluated in this work is a non-motorized tangible rehabilitation platform that therapists can configure according to each patient’s specific needs. Its architecture combines a handle equipped with a red–green–blue (RGB) sensor and colored targets arranged on a height-adjustable table, allowing therapists to adapt the same hardware and game to different motor demands and therapeutic goals by modifying the position, size, sequence, and spacing of the targets. The system also integrates 17 serious games for motor, cognitive, and hybrid motor-cognitive training, together with multimodal feedback and clinically oriented physical adaptations.

Although the initial motivation for the device was upper-limb rehabilitation after stroke, its current configuration was based on broader neurorehabilitation requirements, including repetitive task-oriented practice, movement demands configurable by the therapist, cognitive-motor training, multimodal feedback, and self-assisted or bimanual use [6,7]. These requirements are highly relevant to stroke rehabilitation but are not exclusive to this condition. Therefore, post-stroke rehabilitation remains the primary intended application scenario, while broader neurological rehabilitation provides the clinical context for the feasibility evaluation performed in the present study.

Preliminary tests reported by Sánchez-Gil et al. [8] provided useful evidence for defining clinical-use requirements, usability constraints, and design opportunities. These insights informed the current configuration of the system. The present work, however, focuses on its implementation and feasibility in a clinical neurorehabilitation setting rather than on the technical development of the system itself.

From a sensing perspective, the contribution evaluated in this study is an event-based architecture that integrates a handle-mounted RGB sensor, local embedded processing, calibration, threshold-based color classification, temporal stabilization, and wireless communication with therapist-configurable physical targets. The resulting target detection events link goal-oriented upper limb movements to serious games without the need for continuous position tracking. By changing the position or size of the targets, therapists can use the same detection mechanism to accommodate different movement directions, reach distances, and end-point placement requirements.

For the purposes of this study, feasibility was defined as the practical ability to conduct repeated, supervised sessions (in conjunction with standard neurorehabilitation) in the intended clinical setting, as well as to identify relevant limitations for a subsequent controlled evaluation. Complementary aspects were considered, assessed through participant retention and adherence to sessions, the delivery of the intervention alongside usual care, technical operability during sessions, usability as reported by participants, and clinical staff perceptions of barriers to integration and implementation [9,10]. Usability as reported by participants was considered the primary indicator of acceptability. Feasibility was distinguished from clinical efficacy. Functional and movement-related outcomes were exploratory in nature and were not used to determine whether the system was feasible.

Consequently, the primary objective was to evaluate the feasibility and acceptability of repeated supervised use of the proposed system, including the operational feasibility of its event-based RGB target-sensing mechanism under the evaluated supervised clinical conditions. The secondary objectives were to describe patients’ motivation and their experience with the game during repeated use, to examine clinical staff perceptions regarding its potential integration into routine rehabilitation, and to explore preliminary intra-individual changes in functional and movement-related outcomes. These secondary and exploratory findings were intended to generate hypotheses rather than establish clinical efficacy.

Accordingly, the study was guided by the following research questions (RQs):

- RQ1. To what extent was repeated supervised use of the proposed system feasible and acceptable in a clinical neurorehabilitation setting?
- RQ2. What preliminary within-subject functional changes are observed after a short gamified program in neurological patients?
- RQ3. How do the clinical staff perceive the proposed gamified device and its potential integration into routine rehabilitation?
- RQ4. What level of motivation and enjoyment does the proposed system generate during repeated use?

## 2. Related Work

This section analyzes gamification-based neurorehabilitation systems for the upper extremities, with a particular focus on post-stroke rehabilitation, as this is one of the most extensively studied areas of application for gamification and gamified motor training. Particular attention is given to non-motorized tangible systems, as they represent the technological category most closely aligned with the system evaluated in this study [7,11].

Commercial gamified systems have demonstrated clinical benefits in post-stroke rehabilitation [7,11], although specialized rehabilitation platforms typically rely on specific hardware and relatively closed hardware and software ecosystems [12]. For example, the Rapael Smart Board focuses on upper limb rehabilitation using games based on virtual reality (VR) and high-precision technology, with positive clinical outcomes [13]. Similarly, ArmAble offers gamified upper limb rehabilitation through an adjustable platform and enables bilateral interaction. Ali et al. [14] reported improvements in arm function using this device. These systems demonstrate the clinical feasibility and technological robustness that commercial rehabilitation platforms can offer. However, procurement and maintenance requirements, specific hardware, and reliance on proprietary ecosystems may continue to affect their scalability in some clinical settings [15,16]. In this regard, cost should be viewed as one of several factors influencing implementation, rather than a general limitation on commercial rehabilitation technologies.

Custom-designed, non-motorized gamified systems represent a complementary technological approach based on tangible physical interaction, multisensory feedback, and hardware created specifically for this purpose. For example, approaches based on interactive tables have demonstrated their advantages for collaborative rehabilitation, although they often focus on a specific task format [17]. Other systems based on graphics tablets or screen-centered interaction have demonstrated ease of use and versatility, but offer less flexibility when reconfiguring therapist-guided spatial tasks [18]. More recent tangible systems have combined electronic pegboards with multisensory feedback; the corresponding studies reported favorable preliminary usability in patients with stroke, although the available evidence remains early-stage and based on small samples [19]. Although these approaches support the potential of accessible tangible interaction, they have generally been evaluated in relatively specific interaction formats or during early-stage validation.

More recent studies further demonstrate the growing interest in combining digital rehabilitation tasks with physical, tangible interaction. Pereira et al. [20] proposed a tangible desktop interface for upper limb rehabilitation following stroke, which combined a horizontal screen, a camera-based tracking system, and real objects manipulated during guided simulation. Their results supported the system’s feasibility, usability, and user engagement, although the study focused on short-term feasibility rather than clinical efficacy. Similarly, Huang et al. [21] developed a gamified, tablet-based tangible digital platform featuring a touch-sensitive goal board and color-coded cylindrical elements to assess attention and hand function following a stroke. The system demonstrated favorable usability and psychometric properties, although its primary objective was assessment and monitoring rather than a longitudinal therapeutic intervention.

Motorized and haptic systems represent a complementary technological approach. For example, Peramalaiah et al. [22] evaluated a game-based robotic manipulation device in children with cerebral palsy, illustrating the potential role of assisted force during physical interaction. In Parkinson’s disease, Bektic et al. [23] studied virtual reality games combined with a physical haptic device and suggested that adjustable haptic resistance could help stabilize upper limb motor performance. More generally, robot-assisted rehabilitation can employ adaptive strategies based on providing assistance as needed while encouraging active patient participation.

Rehabilitation robots also serve as a complement to therapy, since adaptive interaction control allows the level of physical assistance to be adjusted based on the user’s performance. Molle et al. [24] proposed a real-time reinforcement learning strategy for robot-assisted upper limb rehabilitation. Q-learning was used to adapt the radial stiffness and movement execution time based on the user’s kinematic performance and physiological state. In this approach, modulating the interaction stiffness allows the robot to provide greater assistance when the user experiences difficulties, while preserving greater freedom when the movement can be performed successfully, thereby balancing physical support, task difficulty, and active participation. Although the study was validated in healthy participants and subjects simulating impaired motor behavior, rather than in neurological patients, it illustrates how reinforcement learning and adaptive impedance-based control parameters can be used to customize robot-assisted interaction in real time.

These robotic systems, equipped with numerous sensors, offer capabilities that fall outside the intended scope of the system designed and evaluated in this study. Specifically, motorized systems can actively assist the patient’s movement, while continuous position tracking can facilitate trajectory reconstruction and detailed kinematic analysis. In contrast, the proposed system focuses on a non-motorized design space in which customization is achieved primarily through therapist-guided physical reconfiguration and in-game difficulty adjustment. Interaction is based on discrete detection of colored targets, rather than continuous tracking, which reduces hardware complexity while maintaining tangible interaction with the rehabilitation workspace.

Taken together, the systems analyzed include commercial rehabilitation platforms, robotic devices, and custom-designed tangible interfaces, each with different capabilities, limitations, and implementation requirements. In this context, the main contribution of the proposed device lies in the integration of event-based RGB target detection, therapist-guided physical reconfiguration, task adaptation at the game level, and a broad catalog of motor, cognitive, and hybrid (motor and cognitive) tasks into a single, non-motorized tangible platform. The therapist can modify the position, size, spacing, and sequence of the targets without changing the hardware or game content, thereby adapting the direction and required reach distance, as well as the physical and cognitive demands of the task, to the therapeutic context. Unlike robotic or continuous-tracking systems, the proposed system does not provide adaptive mechanical assistance or continuous kinematic reconstruction. Instead, it prioritizes reduced hardware complexity and therapist-driven configurability. Consequently, this study does not aim to establish superiority over robotic, commercial, or continuous-tracking systems, but rather to evaluate whether this tangible, therapist-configurable approach is feasible, usable, and acceptable when applied to neurological patients in a clinical rehabilitation setting.

## 3. Materials and Methods

**Figure 1:**
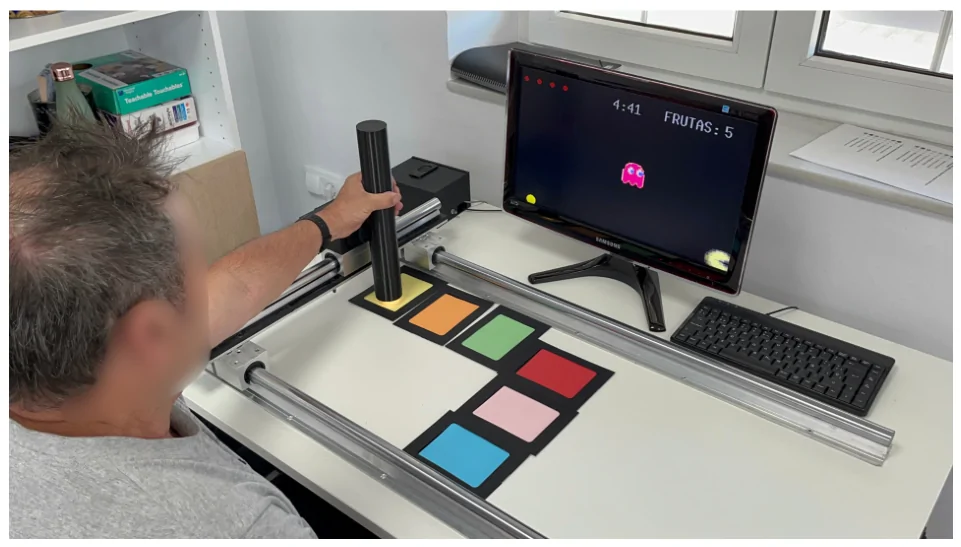
The gamified device deployed in a clinical setting.

This section summarizes the system features required to understand the intervention and describes the clinical feasibility protocol. Extended information on the hardware, firmware, graphical interface, physical development, and serious game design is provided in Supplementary Material S1. Figure 1 shows a patient interacting with the system.

### 3.1. System Features Required for the Intervention

The system consists of two wirelessly connected components: a graphics station that runs the user interface and serious games, and a handle that incorporates an RGB sensor, vibrotactile feedback, a low-friction guided base, and anti-tip guides. Hereinafter, the term “handle” refers to this complete movable unit. Patients move the handle over colored targets placed on a height-adjustable work surface. By changing the position, size, spacing, and sequence of the targets, the therapist can adapt the direction of movement, reach, endpoint-placement demands, and task complexity without needing to modify the hardware or the game.

The games involved selecting targets based on color cues, and the interaction followed a sensor-mediated perception–action sequence. A visual event in the game indicated or was associated with a target’s color. The participant then had to interpret the event, identify the relevant color, locate the corresponding target in the physical workspace, and move the handle toward it. When a time limit was in effect, the target had to be acquired within the game’s specific response interval. A valid RGB detection would then update the game state and trigger the corresponding feedback. In this way, the sensor confirmed the completion of a target-oriented movement, rather than continuously mapping the handle’s movement to an on-screen object.

**Figure 2:**
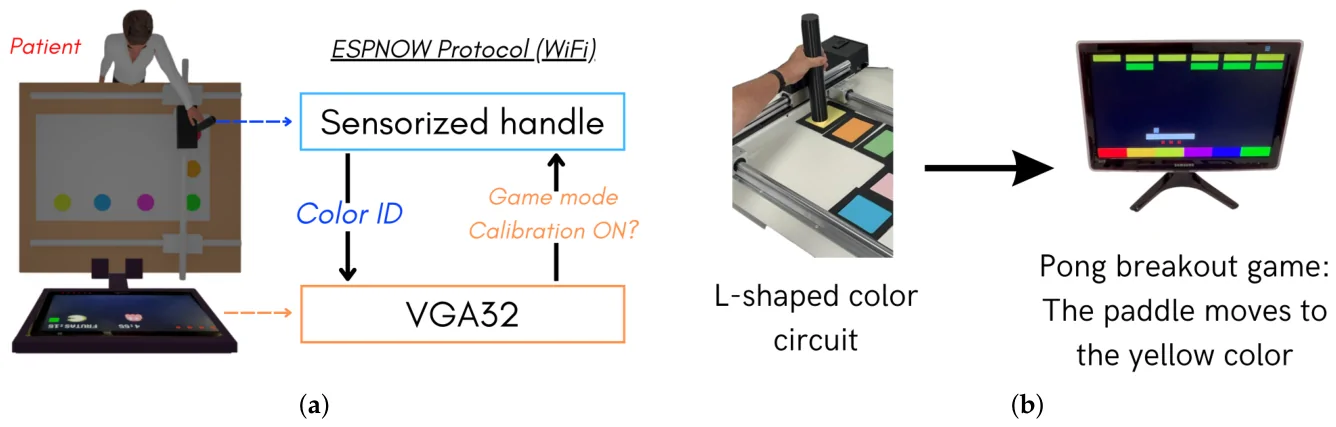
Overall architecture of the proposed system. (**a**) Sensing and Wireless Communication Architecture. (**b**) Overall sensing and game-interaction architecture of the proposed system.

The height-adjustable surface allows for use while sitting, standing, or from a wheelchair. When necessary, movement can be assisted manually by the therapist or self-assisted using the contralateral upper limb. A second handle can be installed for bilateral practice, and the two handles can move independently or be mechanically coupled via a removable connector, so that the less-affected limb assists the more affected one. The coupling is passive and does not provide motorized assistance or assistance-as-needed control. Visual, auditory, and vibrotactile feedback is provided depending on the selected game. These options for customization in adapting the implemented games were intended, as Baranyi suggests in [25], to support and improve the design. Supplementary Material S1 provides more detailed information on the mechanical and electronic aspects. The overall architecture of the system is shown in Figure 2.

#### 3.1.1. RGB Sensing, Signal Processing, and Calibration

Interaction with the physical workspace was achieved through a TCS34725 RGB sensor (Hongkong Yingli International Trading Co., Limited, Hong Kong, China) integrated into the handle. Its readings were processed locally by an ESP32 NodeMCU Dev Kit C V2 development board with CP2102 (AZ-Delivery Vertriebs GmbH, Deggendorf, Germany). During calibration, samples of each reference color were taken for 5 s, and the average of the resulting RGB values was calculated to obtain a representative reference for each color class, which was stored in nonvolatile memory.

**Figure 3:**
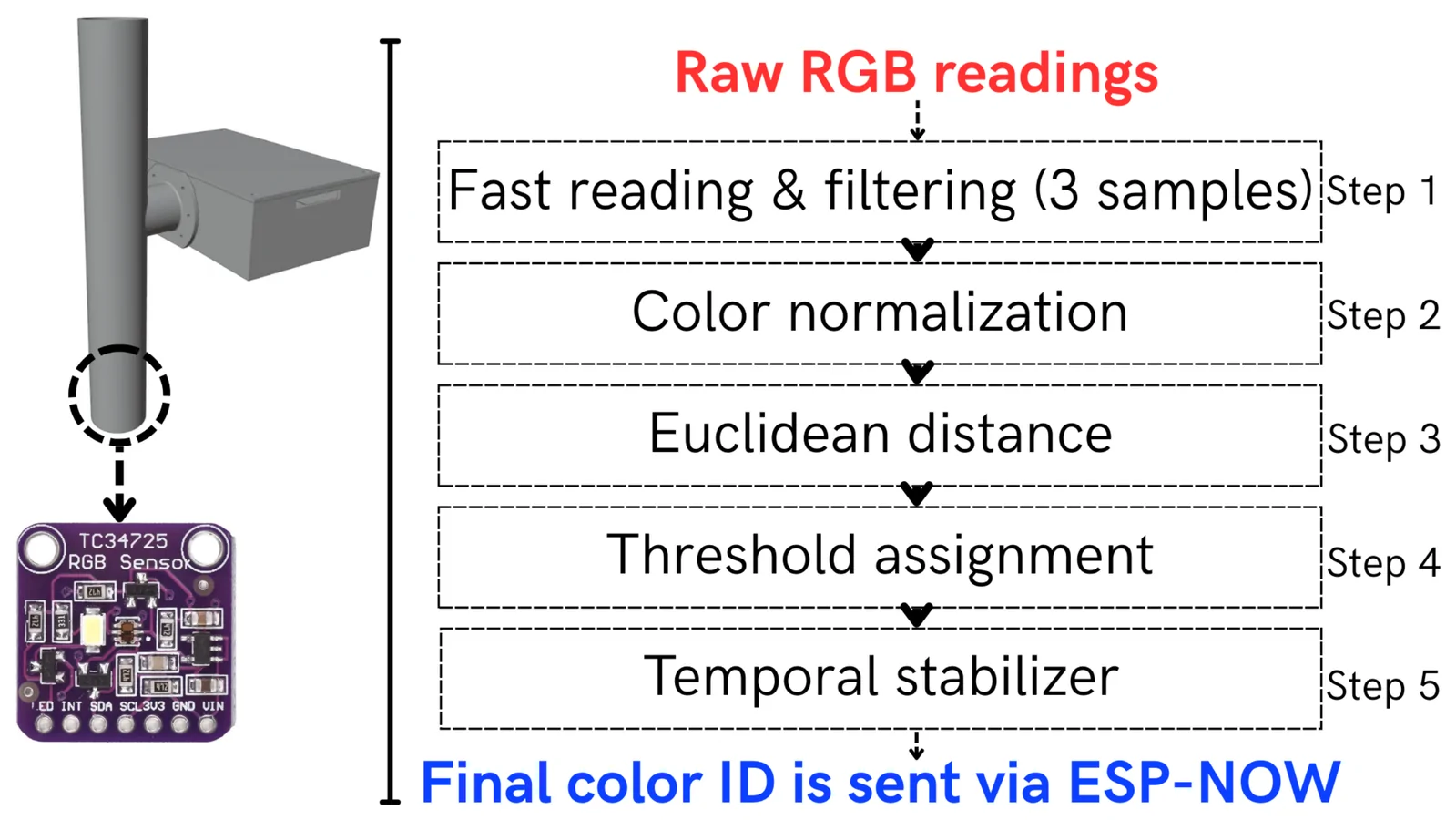
Five-step RGB target-detection pipeline used by the handle during patient–game interaction.

During interaction, the five-step process shown in Figure 3 consisted of: (1) acquiring and filtering three consecutive RGB readings; (2) normalizing the filtered RGB values; (3) calculating the Euclidean distance between the normalized measurement and each stored color reference; (4) threshold-based assignment of the closest valid color class, with readings falling outside all accepted thresholds classified as “unknown”; and (5) temporal stabilization of the classification. The resulting stable color identifier was then transmitted to the graphics station via ESP-NOW. Unknown classifications did not trigger any in-game events. Therefore, the system generated discrete target detection events rather than continuous position or trajectory data. Further details on the firmware, calibration, and signal processing are provided in Supplementary Material S1.

#### 3.1.2. Serious Game Catalog and Motivational Framework

The system includes 17 serious games grouped into the categories of basic, cognitive, and hybrid (motor–cognitive). Basic games promote familiarization and low-complexity interaction. Cognitive games emphasize attention, memory, inhibition, or executive processing. Hybrid games combine upper-limb movement with visuomotor or cognitive demands. This SG variant was designed to address the problem of patients becoming accustomed to long-term gamified therapy, as noted by Xu et al. [26]. Since the games use color cues to select targets, the interaction itself may also require visual attention, the association of stimuli with responses, spatial search, response selection, and (when time limits are in place) movement planning under time constraints. These were task demands inherent in the sequence of interaction and were not treated as measured cognitive effects.

Therapists selected the game and difficulty level based on each participant’s abilities, and some games adjusted their internal difficulty based on the patient’s performance. This level of performance-based personalization has been observed in other studies, such as that by Mubin et al. [27]. This adaptation did not provide mechanical assistance. The motivational design was informed by Self-Determination Theory, particularly the psychological needs for autonomy, competence, and relatedness by Ryan and Deci [28], and by the GameFlow model, which includes concentration, challenge, player skills, control, clear goals, feedback, immersion, and social interaction by Sweetser and Wyeth [29]. These frameworks have been widely used in the literature on the design of serious games for gamified rehabilitation [30,31,32]. The complete catalog of games and their mapping to the design frameworks are included in Supplementary Material S1 and Supplementary Video S1.

### 3.2. Study Design

This was a prospective, non-randomized pilot feasibility study conducted with neurological patients undergoing neurorehabilitation at the Instituto de Neurociencias, Hospital Cruz Roja (Córdoba, Spain). The study evaluated the repeated, supervised use of the proposed system in conjunction with standard care. The clinical cohort constituted the only analytical sample presented in the main article. Before clinical deployment, two preliminary support samples were used only for staged technical and usability refinement. This recommendation to involve users and staff from the early stages in refining the prototype is highlighted by Brassel et al. [33]. Their procedures and descriptive findings are reported in Supplementary Material S3 and were not included in the clinical feasibility analysis.

### 3.3. Participants and Recruitment

Neurological patients were prospectively recruited using convenience sampling at the hospital. The sample size was determined based on the availability of eligible participants during the study period and compatibility with routine clinical care. Participants were required to be clinically stable, capable of providing valid informed consent (either directly or through a legal representative), able to follow simple instructions with whatever support the clinical team deemed appropriate, and deemed safe for participation by the healthcare professionals responsible for their care.

Participants were assigned the anonymized codes ${\mathrm{IN}}_{1}$–${\mathrm{IN}}_{13}$. Thirteen patients began the intervention, and 11 completed the six scheduled sessions, thus constituting the sample for post-intervention analyses and questionnaires. Participant flow, reasons for dropout, and baseline characteristics are described in Section 4.

For descriptive purposes, neurological chronicity was classified according to the time elapsed since the initial neurological episode or injury. Participants were classified as acute when assessed between 1 and 7 days after onset, subacute when assessed more than 7 days and no later than 6 months, and chronic after 6 months [34]. These operational categories were applied consistently when describing neurological participants.

### 3.4. Intervention Protocol

Participants were scheduled to complete six supervised sessions, held twice a week for three weeks, in addition to their usual care. Baseline assessments were conducted before the first session, and post-intervention assessments were conducted after the sixth session.

Each scheduled session lasted approximately 30 min and typically combined up to 5 min of basic games, about 15 min of hybrid motor–cognitive games, and up to 10 min of cognitive games. These time allocations were adjusted as needed to accommodate transitions, brief breaks, the exploratory assessment protocol, and individual tolerance. The therapists selected specific games, difficulty levels, and physical target configurations based on each participant’s motor and cognitive abilities, fatigue level, and performance. No fixed progression was imposed from one session to the next.

After each session, the supervising clinician or researcher completed a structured log documenting attendance, assistance provided, breaks, restarts, adjustments to difficulty or goal settings, calibration requirements, and notable technical or clinical incidents.

### 3.5. Outcome Measures

Outcome measures were grouped into feasibility and usability outcomes, secondary outcomes, and exploratory outcomes based on their role in the pilot feasibility study. Table 1 summarizes the complete assessment schedule for the main clinical cohort. The frequency of assessments varied intentionally depending on the objective of each measure. Validated Spanish versions were used when available. The IMI and iGEQ were administered using study-specific Spanish translations that had not undergone formal cross-cultural psychometric validation.

Brunnstrom staging had initially been considered as a potential exploratory clinical descriptor but was not collected systematically across the clinical cohort. It was therefore not included as an analyzed outcome in the present study.

#### 3.5.1. Feasibility and Usability Outcomes

Feasibility was assessed descriptively across five complementary domains: (1) participant retention and session adherence; (2) intervention delivery alongside usual care; (3) technical operability during sessions; (4) participant-reported usability; and (5) implementation in routine clinical practice. Retention was calculated as the number of participants who completed the six-session program divided by the number who began the intervention. Session adherence among completers was calculated as the number of sessions attended divided by the 66 sessions planned for the 11 participants who completed the program.

The implementation of the intervention was characterized by the total number of sessions provided to all participants who began the intervention, the scheduled duration of the sessions, whether the sessions were delivered alongside usual care as planned, and the number of sessions that ended prematurely due to technical failures. Detection performance and technical operability were evaluated based on session logs, including the number of sessions that required recalibration of the RGB sensor and any technical or detection incidents that affected or prevented the completion of the session. This operational evaluation assessed whether the detection process could withstand repeated supervised interactions under the clinical conditions evaluated. It did not constitute a formal ground-truth evaluation of color-classification accuracy. No quantitative thresholds for progress, a composite feasibility score, or a formal “go/no-go” criterion had been specified a priori. Therefore, the domains were interpreted collectively, and the conclusion regarding feasibility was descriptive and specific to supervised use in the evaluated clinical setting.

Participant-reported usability, treated as the principal acceptability indicator, was assessed using the validated Spanish version of the System Usability Scale (SUS) [35,36]. The SUS consists of 10 items rated on a five-point scale and yields a total score from 0 to 100, with higher scores indicating greater perceived usability.

Implementation in routine care was examined through a brief semistructured interview with the clinical staff who supervised the intervention. The interview addressed integration into routine care, perceived usefulness, patient autonomy, supervision and configuration requirements, and suggested improvements. Responses were summarized descriptively without formal qualitative coding.

#### 3.5.2. Secondary Outcomes

**Participation** was assessed using the Pittsburgh Rehabilitation Participation Scale (PRPS) [37], a clinician-rated single-item measure ranging from 1 (no participation) to 6 (excellent participation). The supervising clinician or researcher completed the PRPS after each session.

**Motivation** was assessed using a study-specific Spanish translation of the 9-item Intrinsic Motivation Inventory (IMI) [38,39]. Items were rated from 1 (not at all true) to 7 (very true) and averaged within the interest/enjoyment, perceived competence, and pressure/tension subscales. Higher scores represent greater intensity of the corresponding construct.

#### 3.5.3. Exploratory Outcomes

Exploratory clinical measures included the Box and Block Test (BBT), Mini-Cog, and Functional Independence Measure (FIM). The BBT score corresponds to the number of blocks transferred with the assessed hand in 60 s, with higher scores indicating greater manual dexterity [40,41]. The Mini-Cog ranges from 0 to 5 and was used exclusively as a brief screening measure. Scores of 0–2 indicate a positive screen for possible cognitive impairment and scores of 3–5 a negative screen [42,43]. The FIM comprises 18 items and provides motor, cognitive, and total scores, with higher values indicating greater functional independence [44,45]. Baseline FIM values were obtained from clinical records.

Gaming experience was explored using a study-specific Spanish translation of the 14-item In-Game Game Experience Questionnaire (iGEQ) [46]. The iGEQ assesses seven components of the experience during gameplay: competence, sensory and imaginative immersion, flow, challenge, tension, positive affect, and negative affect. Items were rated on the original five-point scale: 0 (“not at all”), 1 (“slightly”), 2 (“moderately”), 3 (“fairly”), and 4 (“extremely”). Each component was calculated as the mean of its two corresponding items. The iGEQ was selected because its items focused on the participants’ experience during the gamified activity, rather than on its subsequent effects. The iGEQ was administered immediately after the gamified activity in sessions 2 and 4, rather than during the game, which constituted a non-standard timing of administration. Therefore, its results were interpreted solely as exploratory reports of the gaming experience.

At the end of the final session, participants were asked which elements of the game design they valued most. Participants could also provide spontaneous comments during the intervention, which were recorded in the session observation logs when offered. These comments were not systematically collected, were not provided by all participants, and were used solely as illustrative descriptive information, without formal qualitative analysis.

### 3.6. Exploratory Target-to-Target Movement Analysis

A target-to-target comparison was conducted to generate hypotheses for ${\mathrm{IN}}_{6}$, ${\mathrm{IN}}_{8}$, and ${\mathrm{IN}}_{11}$, the three participants with unilateral disabilities who had the lowest initial scores on the BBT test among those able to complete the standardized protocol. During sessions 3 through 6, participants performed a non-gamified instruction condition and a gamified condition using the same sequence of colored targets. Each condition consisted of nine target-to-target movements, and the order of the conditions alternated across sessions.

Movement time was derived from consecutive target detection events in RGB. The projected one-dimensional displacement, estimated from the video, was measured manually using fixed-camera recordings by reading a white marker located at the distal end of the handle against a fixed ruler. The average speed was calculated separately for each segment as displacement divided by movement time. The results were summarized descriptively, without inferential comparisons. ${\mathrm{IN}}_{10}$ was excluded from this quantitative comparison, as bilateral impairment required an individualized protocol. The complete task sequence, the order of conditions, the video measurement procedure, the aggregation method, the individualized bilateral adaptation, and the session-level data are provided in Supplementary Material S2.

### 3.7. Statistical Analysis

Given the pilot feasibility design, small sample, and clinical heterogeneity, all analyses were descriptive. No confirmatory hypothesis testing, composite feasibility test, or analysis of clinical efficacy was planned. Continuous variables were summarized using means and standard deviations (SD), supplemented by individual values where informative. Categorical variables were reported using counts and percentages. Questionnaire scores were calculated according to their respective scoring procedures. Technical events and staff feedback were summarized descriptively without formal qualitative analysis.

For the exploratory movement comparison, displacement, movement time, and derived mean speed were calculated at segment level and averaged across the nine segments in each participant–session–condition combination. Summaries combining Sessions 3–6 comprised 36 segments per condition and participant. Because the participants were selected purposively and contributed repeated observations, no inferential comparison between conditions was performed.

### 3.8. Ethics and Data Protection

The study protocol was reviewed and approved by the Córdoba Provincial Research Ethics Committee (Comité de Ética de la Investigación Provincial de Córdoba), which is part of the Andalusian Health Service (Servicio Andaluz de Salud, SAS) and is based at the Reina Sofía University Hospital in Córdoba, Spain (protocol PACTUS-241006; SICEIA-2025-003391; committee reference 6376; approval date: 28 January 2026). The committee’s jurisdiction covers the province of Córdoba, where the clinical study was conducted. All procedures were conducted in accordance with the Declaration of Helsinki and applicable data protection regulations, including the European Union’s General Data Protection Regulation (EU GDPR) and Spain’s Organic Law on the Protection of Personal Data and the Guarantee of Digital Rights (Ley Orgánica de Protección de Datos de Carácter Personal y Garantía de los Derechos Digitales, LOPDGDD). Participation was voluntary, and written informed consent was obtained prior to conducting any study procedures, either directly from the participant or, when applicable, from a legal representative. Participants could withdraw at any time without this affecting their clinical care. Specific written consent was also obtained for audiovisual recording and the use of images.

Study data were pseudonymized using cohort-specific participant codes. The correspondence between participant identity and study code was stored separately with restricted access.

## 4. Results

The results pertain exclusively to the clinical cohort and are presented descriptively, in accordance with the pilot feasibility study design. No intervention-related adverse events were recorded. Complete data were available at all scheduled assessment points for the 11 participants who completed the intervention.

### 4.1. Participant Flow and Baseline Characteristics

Thirteen neurological patients began the clinical protocol. Two participants dropped out after the first session. ${\mathrm{IN}}_{12}$ dropped out because the cognitive impairment observed during the interaction prevented him from adequately understanding and recognizing the task instructions. ${\mathrm{IN}}_{13}$ dropped out due to frustration related to upper limb limitations and a preference for focusing on lower-limb rehabilitation. Eleven participants completed the six planned sessions (corresponding to a retention rate of 84.6%) and constituted the sample for the primary analysis. The baseline demographic and clinical characteristics of the participants who completed the study are shown in Table 2.

### 4.2. Operational Feasibility, Sensing Operability, and Usability

The 11 participants who completed the intervention attended all 66 sessions scheduled for them, in addition to receiving their usual care, representing 100% adherence among those who completed the program. Each of the two participants who dropped out completed one session, resulting in a total of 68 intervention sessions delivered and approximately 34 h of scheduled session time. Regarding the operation of the sensors, no technical or detection failures caused the premature interruption of any of the sessions conducted. It was necessary to recalibrate the RGB sensor in 4 of the 68 sessions (5.9%) because changes in ambient lighting affected color detection.

**Figure 4:**
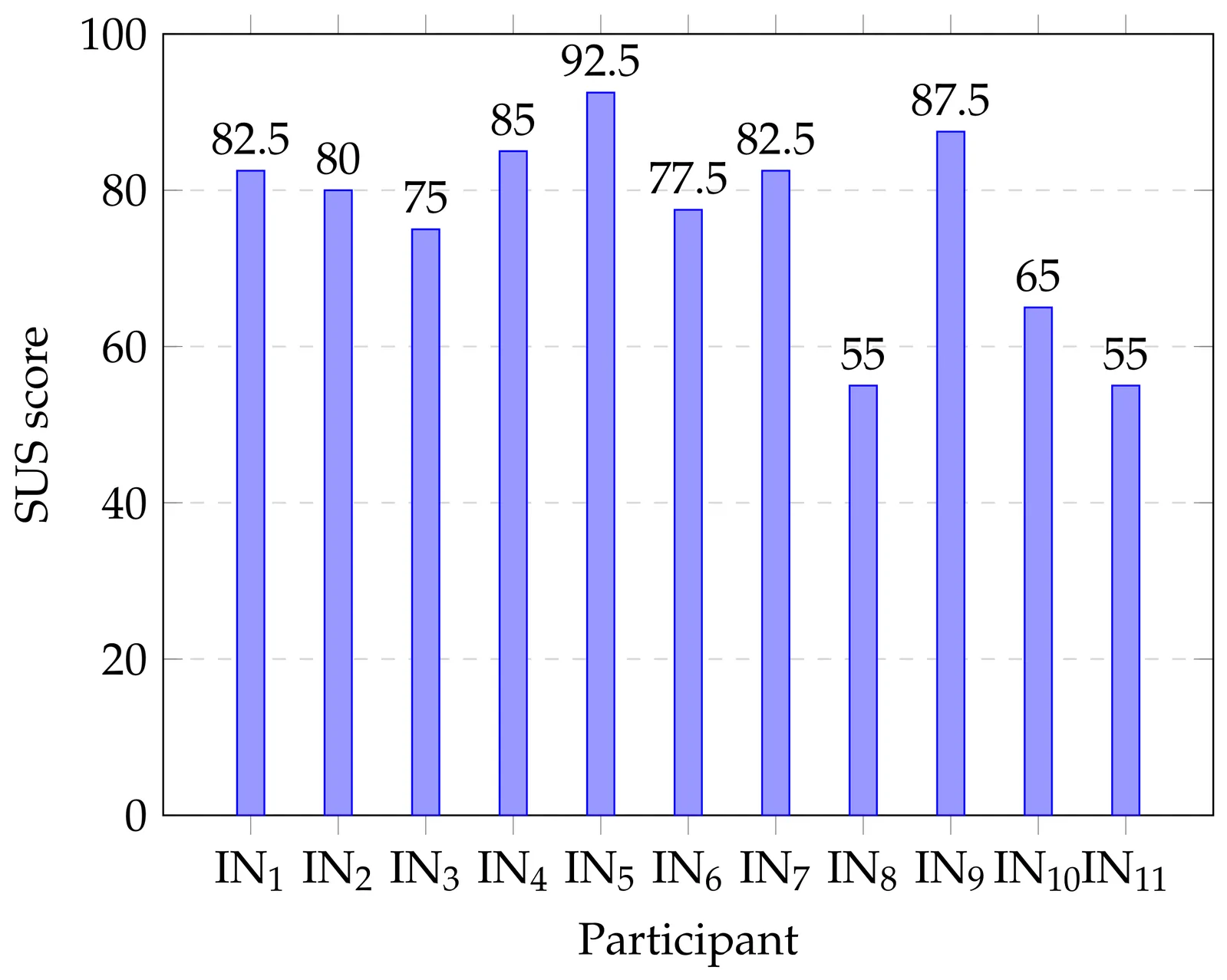
Individual SUS scores for neurological patients in the clinical cohort (*n* = 11).

The eleven participants who completed the intervention achieved a mean SUS score of 76.1 (SD = 12.6), with individual scores ranging from 55 to 92.5. Six participants achieved scores of 80 or higher. Individual SUS scores are shown in Figure 4.

### 4.3. Game Experience, Motivation, and Participation

#### 4.3.1. In-Game Game Experience Questionnaire

The iGEQ was administered after sessions 2 and 4 (non-standard timing). Responses were recorded on the original 0–4 scale, and each component was calculated as the mean of its two corresponding items. The results are summarized in Table 3.

In both assessments, positive affect had the highest overall mean (3.64), followed by flow (3.48), immersion (3.45), and competence (3.32). Challenge scored an overall mean of 2.30 (SD = 0.91), negative affect was 0.16 (SD = 0.26), and tension was 0 in both assessments.

#### 4.3.2. Intrinsic Motivation Inventory

The IMI results for Sessions 2 and 4 are shown in Table 4. Mean interest/enjoyment scores were 6.38 and 6.56, respectively, while mean perceived competence scores were 6.23 and 6.55. The mean pressure/tension score was lower at Session 4 (1.50) than at Session 2 (2.59). Across both assessment points, mean scores were 6.47/7 for interest/enjoyment, 6.39/7 for perceived competence, and 2.05/7 for pressure/tension.

#### 4.3.3. Participation

PRPS scores were recorded after each intervention session. Mean scores ranged from 4.64 to 5.45 across the six sessions: 4.64 (SD = 0.81), 5.18 (SD = 0.87), 5.45 (SD = 0.69), 5.09 (SD = 0.70), 5.09 (SD = 0.54), and 5.36 (SD = 0.81), respectively.

#### 4.3.4. Spontaneous Feedback and Session Observations

Spontaneous comments and observations made during the sessions provided limited contextual information about the participants’ experiences. ${\mathrm{IN}}_{5}$, ${\mathrm{IN}}_{9}$, and ${\mathrm{IN}}_{11}$ described the games as mentally stimulating or as activities that required paying attention simultaneously to movement and to what was happening on the screen. During Session 4, ${\mathrm{IN}}_{3}$ initially appeared distressed and reluctant to exercise, but became engaged once the games began and subsequently continued with the rest of the rehabilitation activities. ${\mathrm{IN}}_{5}$ and ${\mathrm{IN}}_{9}$ stated that the games encouraged them to persevere or to complete the remaining exercises. ${\mathrm{IN}}_{8}$ requested additional games after the scheduled activity in sessions 5 and 6, while ${\mathrm{IN}}_{5}$ requested additional sessions and expressed interest in using the system at home. Since these observations were not systematically collected or formally analyzed, they are included solely to provide illustrative context.

### 4.4. Exploratory Functional Outcomes

#### 4.4.1. Box and Block Test

Table 5 shows the individual BBT scores before and after the intervention for the affected upper limb. Increases were observed between baseline scores and post-intervention scores in most of the affected limbs. Participant ${\mathrm{IN}}_{10}$ had bilateral involvement, so the data for the right and left upper limbs are reported separately.

#### 4.4.2. Mini-Cog

In the initial assessment, six of the eleven participants (55%) scored between 0 and 2, and five (45%) scored between 3 and 5. In the post-intervention assessment, two participants (18%) scored in the range of 0 to 2, and nine (82%) scored in the range of 3 to 5.

### 4.5. Exploratory Target-to-Target Movement Findings

The standardized comparison included ${\mathrm{IN}}_{6}$, ${\mathrm{IN}}_{8}$, and ${\mathrm{IN}}_{11}$. In sessions 3 through 6, according to the descriptive analysis, the mean movement time was shorter and the mean speed was higher in the gamified condition than in the instructional condition for all three participants, while the projected displacement estimated from the video showed relatively small differences between the two conditions. No inferential comparisons were performed. ${\mathrm{IN}}_{10}$ was not included in the standardized quantitative comparison because bilateral upper limb impairment required an individualized protocol. Supplementary Material S2 provides complete summaries at the session and participant levels, along with the individualized bilateral adaptation.

### 4.6. Clinical Staff Feedback

Two physical therapists who directly supervised the use of the system at the hospital completed the post-study semistructured interview. Both noted that the system complemented the motor exercises routinely performed in the rehabilitation room and viewed the incorporation of cognitive tasks and the variety of “serious games” positively. In addition, they observed greater patient participation and motivation.

Both physical therapists also cited the need for continuous or frequent supervision during the setup and selection of games as a limitation to implementation. Other areas for improvement include additional padding around the handle base, better protection of the RGB sensor from ambient light, and the possible future addition of an interaction peripheral for the lower extremities.

### 4.7. Patient Preferences Regarding Game Elements

**Figure 5:**
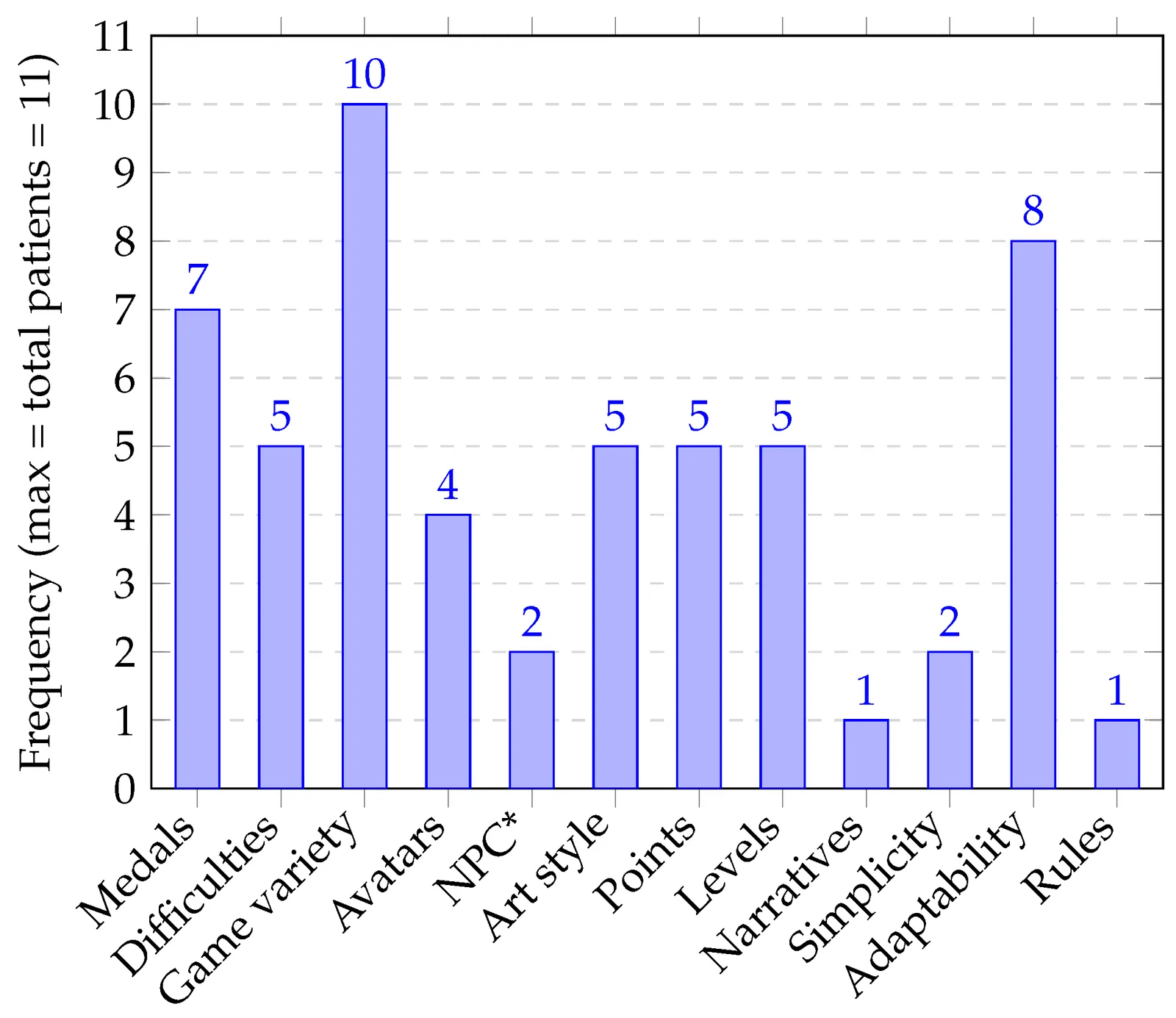
Number of completers selecting each preferred game-design element. *NPC: Non-Playable Characters.

At the end of Session 6, the eleven participants who completed the intervention were asked which elements of the game design they preferred (they could choose up to 5 items). Rodrigues et al. [47] emphasized that “one size does not fit all,” so identifying which elements of the games were most important to the patients could be useful for future designs. The frequency with which each element was selected is shown in Figure 5. ${\mathrm{IN}}_{7}$ additionally associated the arcade-style visual design with games played earlier in life. This individual observation is reported only to illustrate heterogeneity in aesthetic preferences.

## 5. Discussion

The results support a cautious conclusion that the system was feasible and, overall, acceptable for repeated and supervised use as a supplement to standard neurorehabilitation in the hospital setting evaluated. This conclusion is based on combined evidence from participant retention and session adherence, intervention implementation, technical operability, perceived usability, and the perspectives of clinical staff. It is not based on exploratory clinical or movement-related outcomes. This evidence also defines the limits of feasibility, particularly the need for patient selection, therapist supervision, and greater resistance of the RGB sensor to changes in ambient lighting.

### 5.1. Interpretation in Relation to the Research Questions

#### 5.1.1. RQ1: To What Extent Was Repeated Supervised Use of the Proposed System Feasible and Acceptable in a Clinical Neurorehabilitation Setting?

The question of feasibility can only be answered in the affirmative within the context of the controlled conditions and the brief intervention period studied. The results suggest that the protocol could be integrated with usual care for participants who completed it, and no sessions had to be terminated due to a technical failure. The mean score on the System Usability Scale (SUS) exceeded the reference score of 68, but the dispersion of individual scores suggests that the cohort mean should not be interpreted as evidence of uniformly favorable usability [48]. The two premature dropouts and the practical limitations noted by the staff also preclude extrapolation to independent use, to all neurological patients, or to larger-scale implementation. Furthermore, since progression thresholds were not specified a priori, feasibility was assessed descriptively rather than through a formal “go/no-go” rule.

The acceptability of tangible interaction observed in this study is consistent with recent studies on systems for post-stroke patients that combine digital tasks with real objects or physical targets [20,21]. Direct comparison is limited by differences in hardware, study populations, exposure, and study objectives. Nevertheless, these studies support the clinical relevance of maintaining physical interaction rather than limiting rehabilitation to conventional data entry via a screen. The present study provides evidence from repeated sessions in which the therapist physically reconfigured the same interface for participants with heterogeneous abilities.

Implementation limitations are equally important. Reviews on gamified neurorehabilitation and assistive technology for the upper extremities emphasize that clinical adoption depends not only on reliable performance but also on ease of configuration, adjustment, monitoring, and compatibility with the needs of users and clinicians [7,16]. In this regard, configurability by the therapist was both a facilitating feature and a source of workload. Therefore, the system proved viable as a supervised adjunct in this context, but further refinement of the workflow is needed before a broader or less supervised deployment can be considered.

#### 5.1.2. RQ2: What Preliminary Within-Subject Functional Changes Are Observed After a Short Gamified Program in Neurological Patients?

The descriptive increases observed in BBT scores are consistent with meta-analytic evidence indicating that serious game interventions may promote upper limb rehabilitation following a stroke [49]. However, the overall evidence is not consistent: a randomized trial in patients with subacute stroke did not find that video game training was, in general, superior to conventional rehabilitation with the same exercise dose [18]. A recent meta-analysis on virtual reality also associated greater effects with more than 15 h of training administered over 4 to 6 weeks [26]. The present intervention provided approximately three hours of scheduled session time per participant who completed the program, included various neurological conditions, was conducted alongside usual care, and did not include a control group. Therefore, the pre- and post-intervention changes do not allow for distinguishing the effect of the intervention from that of concurrent rehabilitation, spontaneous recovery, habituation, or measurement variability.

Cognitive observations require the same caution. A recent systematic review and meta-analysis suggested that virtual reality interventions may affect certain cognitive domains following a stroke, but the results varied depending on the measures and protocols used and were less consistent with regard to general cognition [2]. In this study, the Mini-Cog was used as a screening tool rather than as an outcome measure designed to detect changes. In addition, some games incorporated visual attention, stimulus-response association, spatial search, and response selection within the sequence of movements. These task demands were neither isolated nor quantified, and the spontaneous comments from some participants describing the games as mentally stimulating or demanding should not be interpreted as evidence of cognitive improvement.

The shorter times between targets and higher projected mean speeds observed in the movement subgroup of three participants also give rise to new hypotheses. An exploratory review revealed that game-derived temporal and kinematic variables can complement clinical assessment, while highlighting the need to validate them against established measures and to conduct better-controlled studies [50]. Since the present estimates were based on end-point timing and one-dimensional manual measurements from videos (without continuous trajectory reconstruction or randomized condition order), they cannot establish a kinematic benefit or identify a mechanism. Their value is limited to serving as a basis for a future controlled movement protocol.

#### 5.1.3. RQ3: How Do the Clinical Staff Perceive the Proposed Gamified Device and Its Potential Integration into Routine Rehabilitation?

Clinical staff considered the system to be a potentially useful complement to conventional rehabilitation, although they noted that the configuration, supervision, and ergonomics posed limitations to its routine use. This combination of perceived clinical value and implementation burden aligns with the design literature, which places therapists at the center of serious game adaptation and, at the same time, identifies quick setup, intuitive operation, comfort, and individualized adjustments as prerequisites for adoption [25,27,33]. Therefore, configurability should not be considered an unconditional advantage: each additional setting may enhance personalization but also consume clinical time.

The practical implication is to maintain therapist control while reducing unnecessary steps in device setup. Patient profiles, reusable presets for sessions, and improvements in ergonomics and sensor isolation could reduce the workload without compromising the system’s intended interaction function. This distinction between technical operation and integration into the clinical workflow is important, as implementation and scalability remain under-studied obstacles in gamified neurorehabilitation, as observed by Tosto-Mancuso et al. [7].

#### 5.1.4. RQ4: What Level of Motivation and Enjoyment Does the Proposed System Generate?

The convergence of measures of motivation, gaming experience, and participation indicates a positive short-term experience, but does not prove that gamification has fostered participation or that it will maintain adherence beyond six sessions. This interpretation is consistent with the design logic of serious games, which use clear objectives, feedback, achievable challenges, and meaningful interaction to foster engagement, while the evidence linking specific game elements to adherence remains incomplete [7,27,32].

The spontaneous comments and observations made during the sessions, which are concisely summarized in Section 4, provide context by showing that some participants perceived the games as mentally stimulating or wanted to continue playing. Aramaki et al. [51] described similar motivational narratives in a small feasibility study on patient-centered virtual reality following a stroke. In both cases, these narratives help identify experiences worth examining but do not allow for the quantification of an effect. In the present study, comments were neither systematically collected nor formally analyzed. Consequently, they do not provide evidence of changes in mood, cognition, or treatment adherence, and a short-term novelty effect cannot be ruled out.

### 5.2. Positioning the System Within Current Neurorehabilitation Technologies

Upper-limb rehabilitation technologies include tangible interfaces, camera systems, or wearable sensors; immersive virtual reality; haptic devices; and motorized robots, each of which offers a different balance between assistance, measurement, feedback, portability, and implementation requirements [11,52,53]. Therefore, the relevant comparison does not lie in whether one category is inherently more accessible or effective, but rather in what capabilities are required for a specific therapeutic goal and clinical context.

Motorized robotic and haptic systems can provide active assistance or resistance and can use continuous force or position data to adapt the interaction and quantify performance [22,23,24]. The proposed system cannot offer these functions. Its detachable connector allows the user to assist themselves using the contralateral limb, but this passive mechanical coupling does not equate to assistance tailored to the patient’s needs during task performance, nor to impedance control. Eliminating motors reduces mechanical and control complexity, but makes the platform unsuitable when externally generated assistance, controlled resistance, or validated continuous kinematic measurements are required.

Camera-based and immersive systems can offer more comprehensive spatial tracking and virtual environments, and recent data support their potential for upper limb training, although results depend on patient characteristics, intervention intensity, and protocol design [26,53]. They may also pose challenges related to tracking conditions, equipment tolerance, or the disconnect between virtual and physical interaction [52]. In contrast, the proposed system maintains contact with real targets and uses discrete color detection events, but it cannot reconstruct motion trajectories, and its optical detection remains, at times, sensitive to ambient lighting.

Conceptually, the system most closely resembles tangible approaches that link physical objects or targets to digital tasks [20,21]. Its sensing contribution lies in using discrete RGB target-acquisition events to connect game cues with goal-directed movements toward therapist-configured physical targets, rather than continuously mapping handle position to an on-screen object. Repositioning and resizing the targets allowed therapists to alter movement direction, reach distance, endpoint-placement demands, and spatial-search requirements without changing the sensing hardware. This design preserves tangible interaction through a low-complexity event-detection pipeline. Its trade-offs are therapist-dependent setup and the absence of continuous kinematic data. The contribution therefore lies in integrating and clinically deploying event-based RGB sensing within a configurable tangible interface, rather than in introducing a new sensor or replacing robotic, immersive, or validated kinematic systems.

### 5.3. Therapist-Guided Personalization and Motivational Design

The ability to modify the physical target layout, select games, and adjust the difficulty level is consistent with the literature on serious games for stroke, which emphasizes meaningful feedback, graded challenges, recoverable failure, and adaptation to motor and cognitive abilities [25,27]. Participants’ differing preferences regarding usability, difficulty level, and design elements reinforce the practical need for adjustments, rather than using a single fixed configuration. However, research on personalized gamification has yielded mixed results, and its superiority over non-personalized approaches cannot be taken for granted [47]. The present study did not compare personalization strategies and, therefore, only demonstrates that therapist-guided adaptation could be implemented.

Self-determination theory, as described by Ryan and Deci [28], and GameFlow proposed by Sweetser and Wyeth [29] served as design frameworks rather than as explanatory models tested in this study. The choice of games and the achievable difficulty level were intended to foster autonomy and competence, while clear goals, immediate feedback, focus, and challenge align with the GameFlow criteria [30,31,32]. The favorable ratings regarding the experience are consistent with these principles but do not validate the proposed mechanisms. Component-level or factorial studies would be necessary to determine whether variety, rewards, feedback, or adaptation independently contribute to motivation or participation.

### 5.4. Limitations of the Study

The small convenience sample was heterogeneous in terms of diagnosis, disease chronicity, motor disability, and cognitive status, and did not include any participants in the acute phase. The study lacked a randomized comparison group. Participants continued to receive usual care. Exposure was limited to six sessions over three weeks, and there was no follow-up. Consequently, no conclusions can be drawn regarding either efficacy or specific effects related to diagnosis or disease stage.

Several outcomes were self-reported or assessed by observers, and floor or ceiling effects reduced variability on some subscales. The Mini-Cog is a screening tool rather than a measure designed to detect short-term cognitive changes. The study-specific Spanish translations of the Intrinsic Motivation Inventory and the 14-item In-Game Game Experience Questionnaire had not undergone formal cross-cultural psychometric validation. Furthermore, the iGEQ was administered immediately after the game, rather than repeatedly during it. Therefore, these results should be considered solely as exploratory descriptions of the experience.

The comparison of movements included a purposively selected subgroup of three participants. Displacement was manually measured from recordings made with a fixed camera, as a one-dimensional projection onto a ruler. No continuous reconstruction of the trajectory was performed. The order of the conditions was not randomized, and the difficulty of the game increased over the course of the sessions. The resulting estimates cannot be generalized, interpreted as validated kinematic data, or used to attribute differences to gamification.

Patient comments were provided voluntarily by only a few participants and were not systematically collected or formally coded. The staff’s conclusions were drawn from a brief, semistructured interview with two physical therapists at a hospital. Both sources are descriptive and may not be generalizable to other patients, professionals, or settings. From an operational standpoint, the recalibration of the RGB sensor in 4 of the 68 sessions and the need for therapist-guided configuration indicate that technical and workflow challenges persist. The sensor’s operability was inferred from session completion rates, recalibration requirements, and recorded incidents. The accuracy of color classification, false-positive and false-negative rates, response latency, and robustness under systematically controlled lighting conditions were not evaluated against a reference standard. Therefore, the results support operational feasibility under the supervised conditions evaluated but do not constitute a formal validation of the sensor’s performance. Finally, the absence of pre-established progression thresholds means that the conclusion regarding feasibility is descriptive and context-specific, rather than a formal decision to proceed.

Future studies should specify progression criteria in advance, use controlled designs with adequate statistical power, and evaluate more homogeneous diagnostic groups over longer periods. They should combine validated motor and cognitive outcomes with prospective measures of implementation, including time spent by staff, assistance needs, configuration burden, technical incidents, and continued use. Future validation of the sensors should quantify accuracy in color classification, false positives and false negatives, response latency, and robustness under controlled and clinically representative lighting conditions. Continuous position tracking would be necessary if the goal were trajectory-based assessment, while formal qualitative methods would be required to investigate the experiences of patients and healthcare professionals. It is also necessary to establish the safety and feasibility conditions for data transmission for medical monitoring through IoMT.

## 6. Conclusions and Future Work

Under the hospital conditions evaluated, the proposed system appears to be feasible and acceptable for repeated, supervised use as a supplement to standard neurorehabilitation. Its event-based RGB detection architecture was operationally viable under the evaluated conditions, although it remains necessary to formally validate the sensors’ performance and improve their robustness against ambient lighting. Overall, the system was well received in terms of ease of use, motivation, and engagement, while its configurable physical and digital features allowed for supervised adaptation to different motor and cognitive abilities. However, the conclusion regarding feasibility is descriptive and context-specific, and does not establish its suitability for independent use, for all neurological patients, or for large-scale implementation.

Exploratory clinical and movement outcomes cannot be interpreted as evidence of clinical efficacy. Future studies should establish progression criteria prospectively and use controlled designs with adequate statistical power, more homogeneous diagnostic groups, and longer follow-up. Technical development should prioritize simplified configuration, improved ergonomics, and optical robustness, while future sensor studies should quantify the accuracy of color classification and latency under clinically representative lighting conditions.

## Figures and Tables

**Table 1:** Outcome measures and assessment for the primary clinical cohort.

Measure	Purpose	Assessment Time
Participant flow and attendance records	Participant retention, adherence among completers, and total number of delivered sessions	Throughout the intervention
System Usability Scale (SUS)	Participant-reported usability as an acceptability-related indicator	After completion of the intervention program
Pittsburgh Rehabilitation Participation Scale (PRPS)	Clinician-rated participation during rehabilitation	At the end of each of the six sessions
Intrinsic Motivation Inventory (IMI), Spanish 9-item version	Motivation and perceived experience during repeated use	After Sessions 2 and 4
In-Game Game Experience Questionnaire (iGEQ), 14-item	Exploratory report of experience during the immediately preceding gamified activity	Immediately after the gamified activity in Sessions 2 and 4 (non-standard timing)
Box and Block Test (BBT)	Upper-limb manual dexterity	Baseline and post-intervention
Mini-Cog	Brief cognitive screening	Baseline and post-intervention
Functional Independence Measure (FIM)	Baseline functional independence	Baseline/clinical record
Technical and observational logs	Session delivery, technical operability, therapist support, and task adaptation	Every session
Clinical staff interview	Integration into routine care, supervision and configuration requirements, and implementation barriers	After completion of data collection

**Table 2:** Clinical cohort: baseline characteristics and Functional Independence Measure (FIM) scores for completers (*n* = 11). Codes ${\mathrm{IN}}_{x}$ denote “Instituto de Neurociencias” participants.

${\mathrm{IN}}_{x}$	Age (y)	Sex	Etiology (Time Since Injury)	Affected Hand	FIM Motor	FIM Cognitive	FIM Total
${\mathrm{IN}}_{1}$	59	Male	Severe traumatic brain injury (chronic, 2 years)	Left	84	30	114
${\mathrm{IN}}_{2}$	43	Female	Severe traumatic brain injury (chronic, 20 years)	Right	90	26	116
${\mathrm{IN}}_{3}$	78	Female	Hemorrhagic stroke (chronic, 2 years)	Left	64	31	95
${\mathrm{IN}}_{4}$	68	Male	Ischemic stroke (subacute, 1 month)	Left	86	35	121
${\mathrm{IN}}_{5}$	54	Female	Polytrauma/neuropathy (subacute, <6 months)	Right	86	30	116
${\mathrm{IN}}_{6}$	51	Female	Ischemic stroke (subacute, 1 month)	Right	17	33	50
${\mathrm{IN}}_{7}$	61	Male	Brain tumor (subacute, 1 month)	Left	85	30	115
${\mathrm{IN}}_{8}$	78	Female	Ischemic stroke (subacute, 1 month)	Left	17	32	49
${\mathrm{IN}}_{9}$	58	Female	Ischemic stroke (subacute, 1 month)	Left	27	30	57
${\mathrm{IN}}_{10}$	67	Male	Hemorrhagic stroke (chronic, 1 year)	Both	20	24	44
${\mathrm{IN}}_{11}$	80	Female	Ischemic stroke (chronic, 1 year)	Right	35	35	70

**Table 3:** iGEQ 14-item scores (0–4): mean ± SD.

Component	Session 2	Session 4	Overall
Sensory and imaginative immersion	3.32 ± 0.56	3.59 ± 0.49	3.45 ± 0.47
Flow	3.36 ± 0.55	3.59 ± 0.54	3.48 ± 0.49
Competence	3.18 ± 0.72	3.45 ± 0.76	3.32 ± 0.57
Positive affect	3.55 ± 0.42	3.73 ± 0.34	3.64 ± 0.32
Challenge	2.18 ± 0.84	2.41 ± 1.24	2.30 ± 0.91
Negative affect	0.23 ± 0.34	0.09 ± 0.20	0.16 ± 0.26
Tension	0.00 ± 0.00	0.00 ± 0.00	0.00 ± 0.00

**Table 4:** IMI subscale scores (mean, SD, and range) for neurological patients at Sessions 2 and 4.

Subscale	Session	Mean	SD	Range
Interest/enjoyment	Session 2	6.38	0.49	5.4–7.0
	Session 4	6.56	0.38	5.8–7.0
Perceived competence	Session 2	6.23	0.82	4.5–7.0
	Session 4	6.55	0.57	5.5–7.0
Pressure/tension	Session 2	2.59	1.45	1.0–5.0
	Session 4	1.50	0.81	1.0–3.0

**Table 5:** Box and Block Test scores for the affected upper limb before and after the intervention.

Participant	Affected Limb	Pre	Post
${\mathrm{IN}}_{1}$	Left	42	45
${\mathrm{IN}}_{2}$	Right	29	38
${\mathrm{IN}}_{3}$	Left	25	34
${\mathrm{IN}}_{4}$	Left	39	54
${\mathrm{IN}}_{5}$	Right	44	52
${\mathrm{IN}}_{6}$	Right	2	9
${\mathrm{IN}}_{7}$	Left	28	33
${\mathrm{IN}}_{8}$	Left	0	1
${\mathrm{IN}}_{9}$	Left	33	44
${\mathrm{IN}}_{10}$	Both	0 (R)/18 (L)	0 (R)/24 (L)
${\mathrm{IN}}_{11}$	Right	0	8

## Data Availability

The data presented in this study are available on reasonable request from the corresponding author due to ethical and privacy restrictions involving pseudonymized clinical data. The data are not publicly available because participant-level clinical data could compromise privacy under the approved consent and data protection protocol. All reported results are included in the manuscript and Supplementary Materials.

## Supplementary Materials

The following supporting information can be downloaded at https://www.mdpi.com/article/10.3390/s26185956/s1, Supplementary Material S1 provides the extended technical implementation, physical design, serious game catalog, and theoretical design mapping. References [5,7,11,12,15,16,17,18,19,25,27,28,29,30,31,32,52,54,55,56,57,58,59,60,61,62,63,64,65,66,67,68,69,70,71,72,73,74,75,76,77,78,79,80,81,82,83,84,85,86,87,88,89,90,91,92] are cited in Supplementary Material S1. Supplementary Material S2 provides the complete exploratory movement protocol, video-based measurement procedure, session-level summaries, and individualized bilateral adaptation for ${\mathrm{IN}}_{10}$. Supplementary Material S3 reports the procedures and descriptive findings of the preliminary support samples used during system refinement. These samples were not part of the clinical feasibility analysis and are included exclusively for methodological transparency. Supplementary Videos S1 and S2 illustrate the serious-game interaction and individualized bilateral use, respectively.
