# Supplementary material for: A Therapist-Configurable Serious Game System for Upper-Limb Neurorehabilitation: A Pilot Feasibility Study
Source: Sensors (Basel). 2026 Sep 20;26(18):5956. doi: 10.3390/s26185956 (PMC13611459; doi:10.3390/s26185956)
Supplement: Supplementary file 1 [file sensors-26-05956-s001.zip › Supplementary Material S1 - Technical Details.pdf]

Supplementary Material S1: Full Technical and Design Details of the Proposed System

This supplementary material provides an extended description of the technical implementation and design rationale of the proposed gamified neurorehabilitation system. It complements the concise system description provided in the main manuscript by detailing the hardware architecture, sensing and communication subsystems, electronics, graphical interface, power supply, handle development, serious-game catalog, and the theoretical frameworks used to guide game design.

I. System Architecture and Design Requirements

The proposed system (Figure 1) consists of a graphics station and a handle comprising an RGB-sensor-equipped grip, vibrotactile feedback, and a guided low-friction base. Hereafter, "handle" denotes the complete movable unit. The patient interacts with the system by moving the handle over reconfigurable colored targets placed on a height-adjustable work surface. Target position, size, spacing, and sequence can be modified by the therapist to define different movement patterns and task demands without modifying the hardware or the serious game itself.

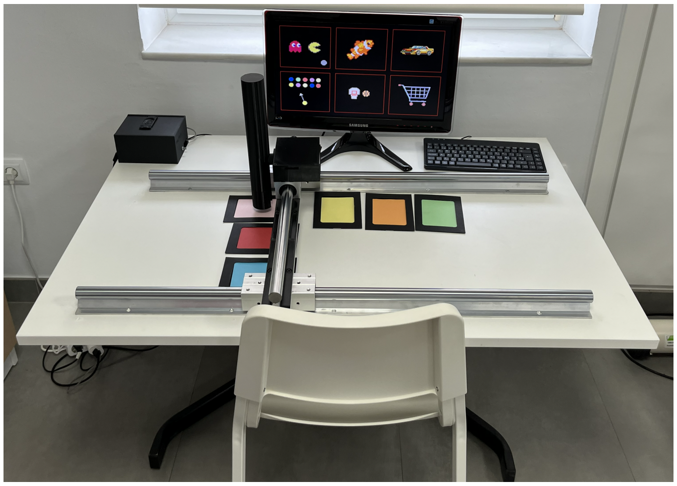

Fig. 1: Overview of the proposed gamified neurorehabilitation system.

A. *Clinical and engineering design requirements*

After reviewing the gaps identified in the literature and the design requirements reported for gamified neurorehabilitation systems, four main priorities were established:

- Lightweight and non-invasive components intended to facilitate installation and use across different clinical environments [1–8].
- Reliable target detection and responsive interaction to support repetitive task-oriented practice aligned with neurorehabilitation principles [9–12].
- Safety and ergonomics through robust hardware and clear interaction mechanics [1, 9, 13–15].
- Multimodal feedback, including visual, auditory, and vibrotactile cues together with post-game performance feedback, to reinforce successful interaction while reducing frustration and maintaining engagement [16–19].

These requirements were intended to support physical, cognitive, functional, and affective aspects of neurorehabilitation, including Activities of Daily Living (ADL), while allowing adaptation to patient abilities and preferences [20–23].

The principal clinically relevant design features were:

1) **Height-adjustable work surface.** The system can be used while standing, seated, or from a wheelchair and can be adjusted according to body dimensions and balance limitations. Among the devices reviewed, a comparable height-adjustment feature was identified in ArmAble [24].
2) **Guided low-friction interaction and assisted use.** The handle is mounted on a low-friction platform with anti-tip guides. This improves stability and allows the affected upper limb to be moved with reduced mechanical resistance. When required, movement can be facilitated by the therapist or self-assisted using the contralateral limb. For bilateral or self-assisted practice, a second handle can be installed on additional linear guides. The two handles can be used independently or mechanically coupled through a removable connector, allowing movement generated by the less-affected limb to assist the more-affected limb. This passive coupling does not provide motorized assistance, force control, adaptive impedance, or assistance-as-needed.
3) **Therapist-directed physical reconfiguration.** Colored targets can be repositioned and resized so that the same serious game can be used with different movement directions, ranges, target spacings, and sequences. Larger targets can facilitate gross reaching tasks, whereas smaller targets increase endpoint-placement precision and visuomotor demands.
4) **Broad serious-game repertoire.** Seventeen games with different motor, cognitive, and combined motor-cognitive demands were implemented to increase task variety and allow selection according to patient abilities and preferences.
5) **Simplified user interface.** A higher-resolution VGA display and keyboard-based navigation were selected to facilitate game selection, calibration, and task control

[8, 13, 14, 25].

6) **Rapid RGB recalibration.** A dedicated calibration state can be activated from the central station when environmental conditions or target cards change. Complete recalibration of all reference colors requires less than two minutes.
7) **Multimodal performance feedback.** Depending on the game, patients receive visual, auditory, and vibrotactile feedback during interaction, together with scores, messages, and performance-based rewards after task completion.

These engineering decisions were intended to link technical implementation directly to therapeutic usability, rather than to introduce isolated hardware features. In particular, therapist-directed target reconfiguration allows game content and required physical movement patterns to be partially decoupled, distinguishing the proposed architecture from systems in which each game is more closely tied to a predefined trajectory or interaction format [22, 24, 26–30].

II. Technical Implementation

A. Hardware architecture and resources

The system contains two wirelessly connected units: the graphics station and the handle.

1) Graphics station: The main components are:

- **TTGO VGA32**: ESP32-PICO-D4-based module with integrated WiFi and Bluetooth connectivity, a 240 MHz dual-core processor, embedded flash memory, and a 40 MHz crystal. It manages the graphical user interface.
- **LCD screen**: 20-inch VGA display with a maximum resolution of 1600 × 900 pixels and a 16:9 aspect ratio.
- **Keyboard**: PS/2 keyboard used for menu navigation, game selection, calibration access, and task interruption. During gameplay, the Escape key allows the task to be stopped immediately.
- **PAM8403 and speaker**: Class-D audio amplifier and speaker used to provide auditory feedback and background music. The audio level can be adjusted or disabled when necessary.
- **DFPlayer Mini**: Stand-alone audio module with microSD support for MP3 and WAV playback.
- **Height-adjustable desk**: 120 × 80 cm work surface with an adjustable height between 68 and 117 cm.

2) handle: The main components are:

- **ESP32**: 32-bit microcontroller with WiFi and Bluetooth connectivity and a dual-core processor. It processes the RGB sensor measurements and manages communication with the graphics station.
- **TCS34725 RGB sensor**: Digital color sensor that measures red, green, blue, and clear-light intensity through an I^2C interface.
- **Vibratory actuator**: Provides vibrotactile feedback during interaction.
- **MP1584EN**: Synchronous buck converter used to regulate the battery supply to the required operating voltage.

B. RGB sensing, signal processing, and calibration

The movement interface consists of a 3D-printed handle attached to bearings running along linear guides. The handle supports the affected upper limb and incorporates the handle used to interact with the colored targets. The RGB target-detection pipeline is summarized graphically in Figure 3 of the main manuscript.

During calibration, each reference color is sampled for 5 seconds. The corresponding red, green, and blue measurements are accumulated and averaged to obtain a representative centroid for each color class. These calibration values are stored in the ESP32 EEPROM so that the references are retained after power-off and reboot.

During gameplay, incoming RGB measurements are filtered and processed before being compared with the calibrated color references. The closest calibrated class is selected according to the distance between the current measurement and the stored reference values, provided that the distance remains below the predefined acceptance threshold for that class. Measurements lying outside all accepted regions are classified as unknown. This prevents arbitrary assignment when the sensor is not located over a valid target or when measurements are excessively affected by environmental illumination, sensor alignment, target variation, or other disturbances.

A temporal stabilization stage is subsequently applied so that a new target identifier is accepted only after the candidate remains stable for a predefined interval. The resulting discrete color identifier is then transmitted to the graphics station and used as the interaction event by the serious game.

The five-step target-detection pipeline comprised: (1) acquisition and filtering of three consecutive RGB readings; (2) normalization of the filtered RGB values; (3) calculation of the Euclidean distance between the normalized measurement and each stored color reference; (4) threshold-based assignment of the closest valid color class, with measurements outside all thresholds classified as unknown; and (5) temporal stabilization before transmission of the resulting color identifier to the graphics station.

Importantly, this sensing architecture provides *discrete target-detection events*. It does not reconstruct continuous end-effector position or movement trajectories. Consequently, variables derived from these detections represent target-to-target interaction metrics rather than continuous kinematic tracking.

When neither gameplay nor calibration is active, the firmware disables RGB acquisition and places the wireless interface in a low-power state.

A visual example of the interaction between the physical target circuit and a serious game is shown in Figure 2.

C. Wireless communication

To avoid a cable between the moving handle and the graphics station, ESP-NOW was used for wireless communication between ESP32 devices. ESP-NOW enables short, low-latency data exchanges in the 2.4 GHz band without requiring conventional network infrastructure.

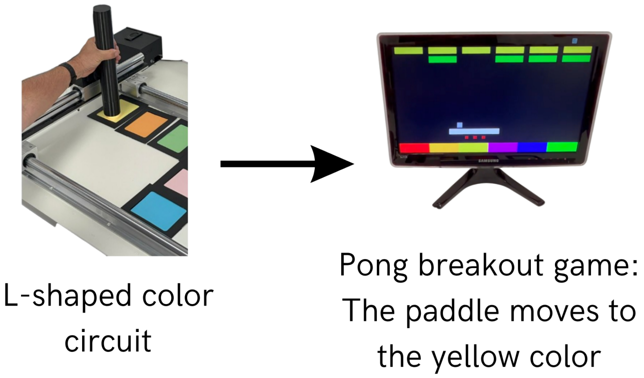

Fig. 2: Example of patient interaction. The physical colored-target arrangement determines the movement sequence, whereas target detections control the corresponding serious-game interaction.

The ESP32 located in the handle processes the TCS34725 measurements locally and transmits the resulting color identifier to the VGA32 station while a game is active. Processing the sensor data locally avoids transmitting continuous raw RGB measurements and reduces wireless traffic and power consumption. In the opposite direction, the graphics station communicates the current operating mode, including game and calibration states.

D. Electronics and printed circuit boards

Custom printed circuit boards were designed for both the handle and the graphics station to reduce wiring complexity and improve integration.

Figure 3 shows the electrical schematic and PCB of the handle. Figure 4 shows the corresponding implementation for the graphics station.

E. Graphical user interface and software architecture

At hardware level, the graphical user interface consists of the VGA32 connected to the VGA display and PS/2 keyboard. The display presents the menus, task instructions, serious games, and post-game information.

The software interface was implemented using the FabGL library (version 1.0.9). A state-machine structure manages navigation between the main menu, difficulty selection, pre-game information, game execution or calibration, and final performance information. At task completion, the interface returns to the main menu.

The audio subsystem includes the DFPlayer Mini, PAM8403 amplifier, and speaker. Decoupling capacitors were incorporated to stabilize the power supply and reduce low- and high-frequency electrical noise.

F. Power supply

The graphics station and handle use independent power architectures.

1) Graphics station: Because the VGA display requires mains power, the station is connected to the electrical supply. A 220 V-to-5 V power adapter is used to supply the VGA32 through micro-USB. A second 5 V output supplies the PAM8403 amplifier and speaker subsystem.

2) Handle: The mobile handle is powered using two 18650 batteries connected in series, providing a nominal voltage of 7.4 V. An MP1584EN buck converter regulates this voltage to a stable 5 V supply for the embedded electronics. The corresponding electrical implementation is shown in Figure 3a.

G. Physical handle and iterative redesign

The physical interaction handle combines the height-adjustable table, linear guides, bearings, the handle carriage, anti-tip structures, and the upper-limb support surface. The handle was refined through two main design iterations before clinical implementation.

Iteration 1 (D1): The first iteration established preliminary dimensions for the graphics-station housing, handle, handle, and anti-tip structures. Figure 5 shows this initial configuration.

Preliminary laboratory testing identified five main limitations:

1) The anti-tip structures did not sufficiently constrain lateral movement of the handle.
2) The handle grip was too small for comfortable use.
3) The handle walls lacked sufficient mechanical rigidity.
4) The support surface was too narrow to comfortably accommodate the affected upper limb.
5) The original 60 cm linear guides did not provide sufficient width for simultaneous installation of two handles for bilateral use.

Iteration 2 (D2): The second iteration addressed these limitations. The handle dimensions were enlarged to improve upper-limb support and accommodate the PCB and battery. The anti-tip guides were redesigned to improve stability, and the handle was widened from 3 cm to 5 cm for users requiring a larger grip.

The graphics-station housing was also modified to facilitate access to internal components and PCB mounting. Finally, the SBR25 linear guides were extended from 60 cm to 100 cm, allowing two handles to be used simultaneously for bilateral or self-assisted training.

This redesign translated observations from preliminary laboratory use into specific improvements in stability, ergonomics, mechanical robustness, and support for bilateral interaction before implementation in the clinical cohort.

III. Serious-Game Catalog and Design Rationale

The system incorporates 17 serious games (SGs) organized into three broad categories: basic, cognitive, and hybrid motor-cognitive games. Table I summarizes the purpose and principal therapeutic elements of each category. The complete game catalog can also be viewed in Supplementary Video 1 and at https://youtu.be/6KjKDYkwnk8.

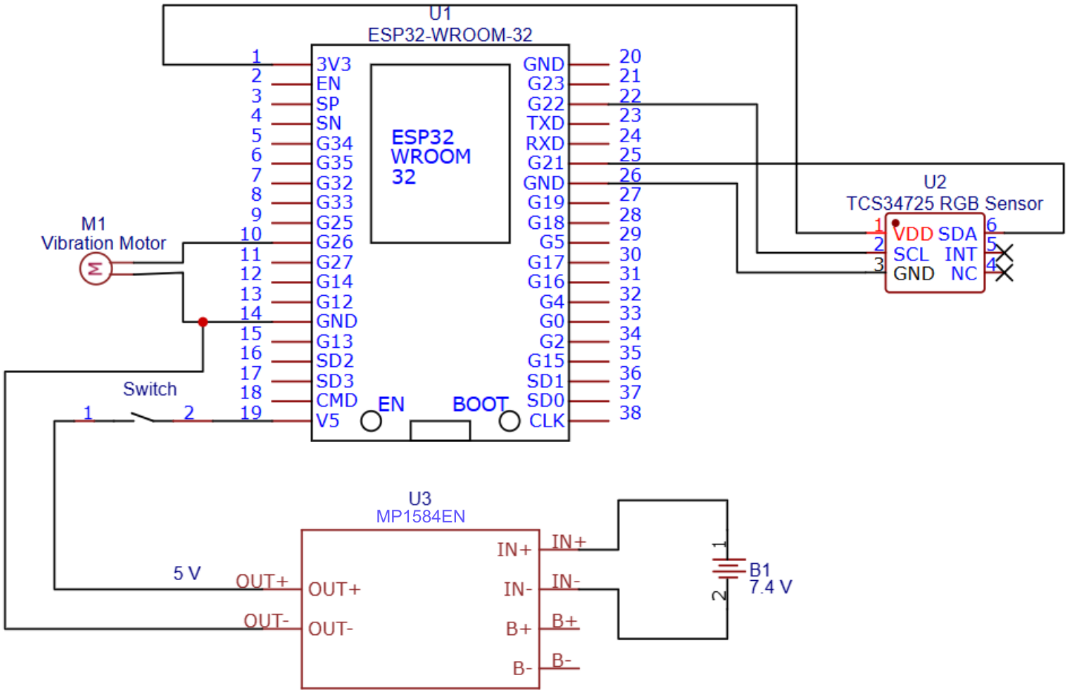

(a) Handle electrical schematic.

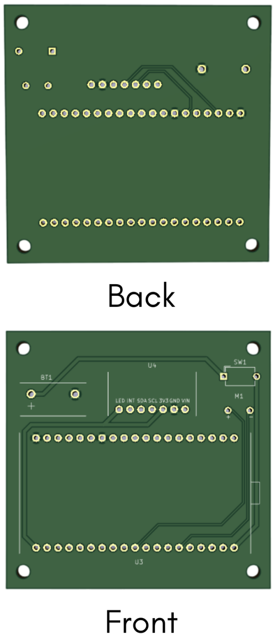

(b) Handle PCB.

Fig. 3: Handle hardware design.

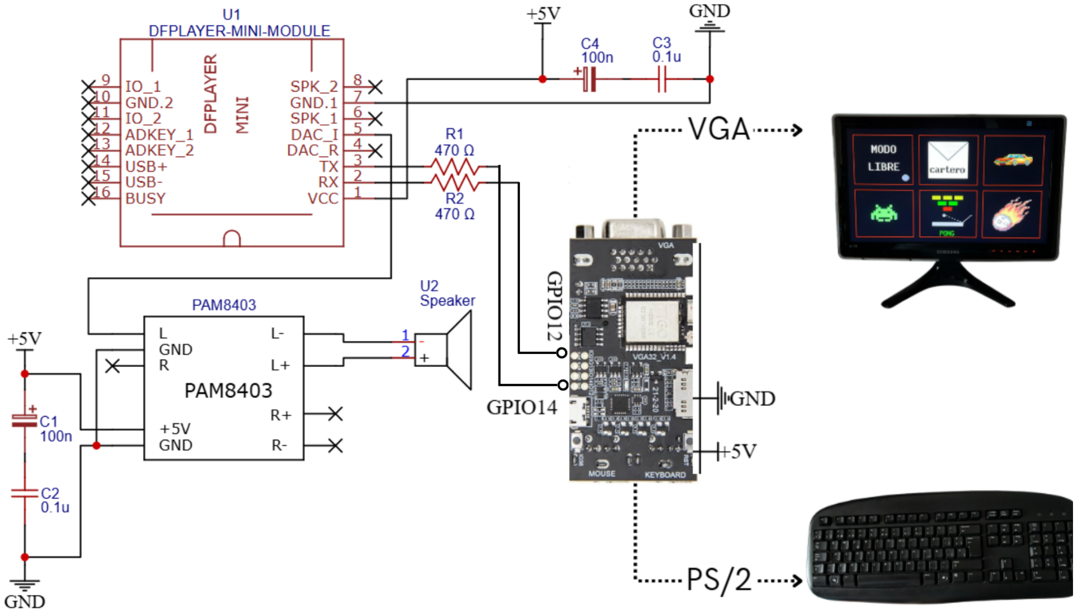

(a) Graphics-station electrical schematic.

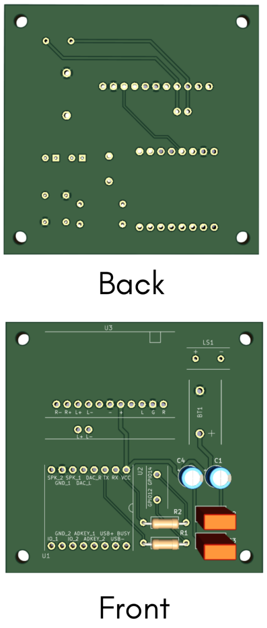

(b) Graphics-station PCB.

Fig. 4: Graphics-station hardware design.

A. *Game design strategy*

The 17 SGs were conceived as a therapeutic catalog rather than as a set of unrelated mini-games. Previous taxonomies and design studies indicate that rehabilitation-oriented SG should consider the target clinical application, functions to be trained, interaction modality, feedback, adaptability, accessibility, and intended context of use [31–34].

Accordingly, the catalog was divided into basic, cognitive, and hybrid games so that therapists could combine different levels of motor and cognitive demand. The variety of themes and interaction styles was also intended to accommodate heterogeneous patient preferences and previous experience with digital games [16, 20, 35].

Basic games provide a low cognitive load, immediate inter-

TABLE I: Classification of the implemented serious games.

**Game type**	**Group description**	**Core gameplay and therapeutic elements**	**Games in this group**
Basic games	Simple tasks with basic rules and no time pressure or enemies. They support familiarization and warm-up and remain accessible for patients with greater cognitive limitations.	Free exploration of the workspace; repetitive reaching; familiarization with target-based interaction and feedback; gross upper-limb control with low cognitive load.	*Free Mode*, *Feeding PACMAN*, *Mailman*
Cognitive games	Games in which the main challenge involves memory, attention, perception, or executive functions, while physical interaction remains comparatively simple.	Short-term and working memory; matching and recall; audiovisual association with ADL-related contexts; sequencing; selective attention; cognitive flexibility; inhibition.	*Memorization cards*, *ADL*, *Stroop*, *Shopping list*, *Simon Says*, *Mystery box*
Hybrid games	Games combining repeated upper-limb movements with additional cognitive and visuomotor demands.	Continuous reaching; rapid target selection; visuomotor coordination; trajectory prediction; obstacle or enemy management; decision-making; divided and sustained attention.	*Aliens*, *Bubble shoot*, *Basket*, *Racing*, *Fishing*, *Asteroids*, *Pong Breakout*, *PACMAN run*

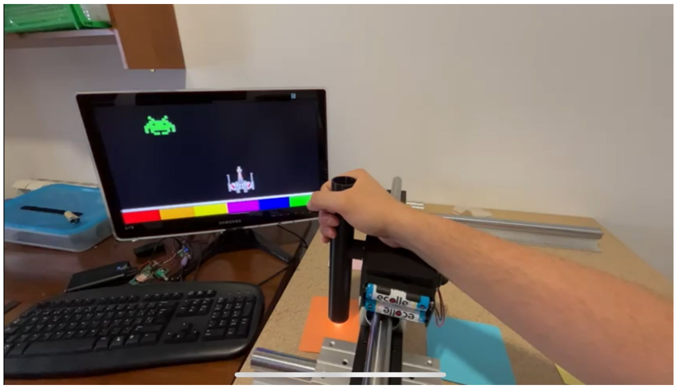

Fig. 5: Initial handle iteration (D1). The handle and upper-limb support surface were subsequently enlarged, and the anti-tip system was redesigned.

action, and relatively accessible success conditions, which can be useful during familiarization or for users with greater limitations [36]. Familiar scenarios and ADL-related themes were incorporated where appropriate to support understandable and recognizable interactions [37, 38].

Cognitive games emphasize processes such as attention, memory, inhibition, cognitive flexibility, and audiovisual association, whereas hybrid games combine repeated physical interaction with additional demands such as rapid target selection, trajectory prediction, time pressure, enemy avoidance, decision-making, or sustained visuomotor attention [31–33, 37].

Recognizable themes and selected interaction conventions from well-known game genres were incorporated when considered useful for familiarity or engagement. These elements were therapeutically reinterpreted rather than intended as direct reproductions of commercial games.

Games provide selectable difficulty levels that can modify parameters such as available response time or the speed of moving elements. Tasks can also be interrupted at any time using the Escape key when fatigue or overload occurs.

Depending on the specific game, feedback can include points, lives, visual indicators, encouraging or congratulatory messages, auditory cues, and bronze, silver, or gold medals according to performance.

B. Game elements and performance-based difficulty adaptation

The principal game-design elements include:

- Selectable difficulty levels to maintain an appropriate challenge and reduce monotony [19, 39, 40].
- Visual, auditory, and vibrotactile feedback [41–44].
- Retro-inspired 2D aesthetics intended to preserve visual simplicity and accessibility [45].
- Game elements including points, awards, time pressure, levels, avatars, lives, goals, Player-versus-Environment mechanics, instructions, and progress indicators [41, 46, 47].
- Performance-driven adaptation of selected *game parameters* in a subset of games [1, 15, 44].

The latter refers specifically to adaptation of the digital game challenge and should not be interpreted as adaptive mechanical assistance or impedance control.

For example, in *Aliens*, enemy firing frequency can increase as the player succeeds. In *Racing*, opponent speed is modified according to relative performance. In the *ADL* game, multiple audio cues are selected without immediate repetition. In *Pong Breakout*, the trajectory of the ball can be adjusted late in the task to avoid excessive repetition when only one block remains. Finally, *Stroop* incorporates Stroop-type interference to increase cognitive demand [48].

IV. Gamification Frameworks

The serious-game design incorporated motivational principles intended to support both perceived competence and sustained engagement. Points, performance awards, graded difficulty, immediate feedback, and selectable game conditions

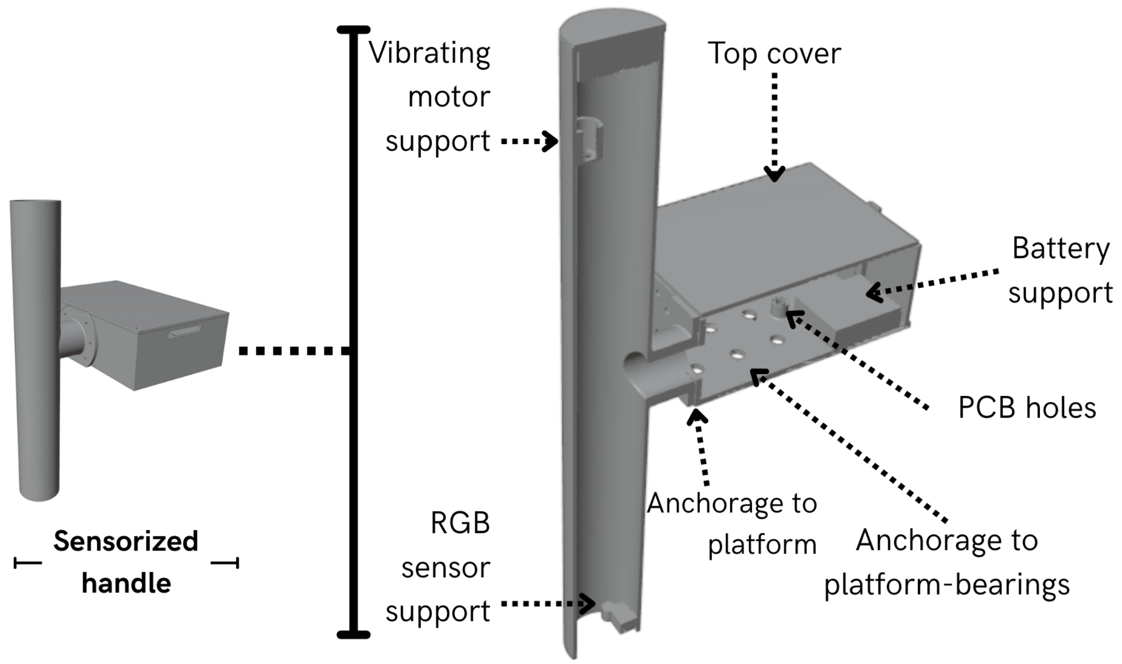

Fig. 6: Final handle design (D2), highlighting its principal mechanical and electronic integration features.

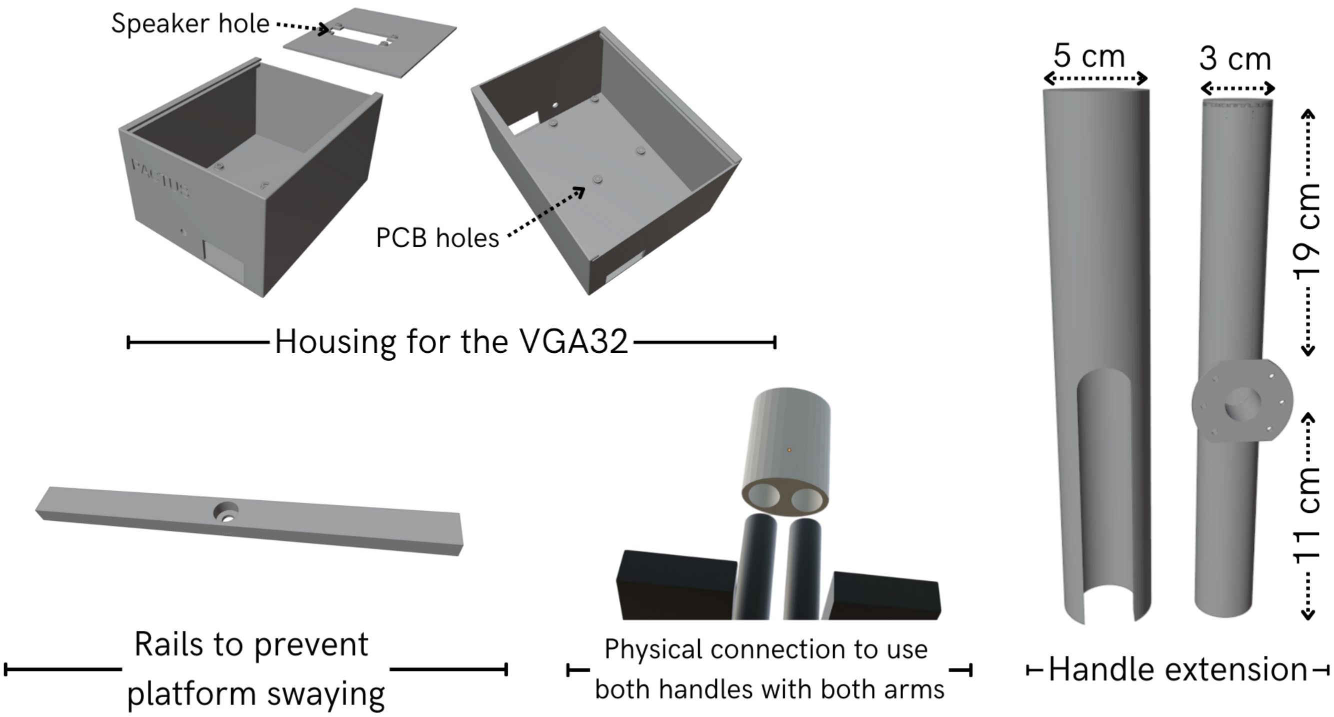

Fig. 7: Mechanical components incorporated into the final system design (D2).

were used to provide clear goals and progression while allowing the therapist to adapt the experience to patient abilities [47, 49–51].

Two frameworks were used principally to organize these design decisions: Self-Determination Theory (SDT), as described by Ryan and Deci [52], and the GameFlow model proposed by Sweetser and Wyeth [53].

A. *Self-Determination Theory*

SDT identifies autonomy, competence, and relatedness as basic psychological needs associated with motivation and well-being [52]. Subsequent studies have applied these principles to customization and adaptive gamification [54–56]. Table II summarizes how these concepts were translated into system and game-design decisions.

TABLE II: Mapping between Self-Determination Theory (SDT) and the proposed system design features.

SDT element	Targeted need / principle	Design strategies and examples
Autonomy	Feeling in control of one's own actions and choices.	1. Selectable difficulty levels and game types.<br>2. Possibility to stop a task immediately.<br>3. Therapist-directed selection of different physical movement patterns for the same game through target reconfiguration.<br>4. Adjustable work-surface height and availability of self-assisted or therapist-assisted interaction when required.
Competence	Feeling effective, skilled, and capable of progressing.	1. Graded difficulty.<br>2. Performance-based awards.<br>3. Clear visual, auditory, and vibrotactile feedback.<br>4. Performance-based adaptation of selected game parameters.<br>5. Basic games designed to provide accessible early interaction for patients with greater impairment.
Relatedness	Feeling connected with meaningful contexts and people.	1. Everyday-life themes and familiar contexts.<br>2. Therapist–patient interaction during supervised sessions.<br>3. Multiplayer interaction was not implemented in the present version.

TABLE III: Mapping between GameFlow elements and design features.

GameFlow element	Targeted need / principle	Design strategies and examples
Challenge–skill balance	Maintaining an appropriate balance between task difficulty and patient ability.	1. Multiple difficulty levels.<br>2. Performance-based adaptation of selected game parameters.<br>3. Basic, cognitive, and hybrid game categories for different challenge profiles.
Clear goals and feedback	Providing explicit objectives and understandable performance information.	1. Clear task objectives.<br>2. On-screen instructions.<br>3. Immediate visual, auditory, and vibrotactile feedback.<br>4. Scores and performance-based rewards.<br>5. Encouraging messages following unsuccessful attempts.
Control and usability	Supporting predictable and manageable interaction.	1. Guided handle.<br>2. Simplified keyboard-based GUI.<br>3. Rapid RGB recalibration.<br>4. Possibility to interrupt tasks immediately.<br>5. Non-wearable tangible interaction.
Concentration and immersion	Supporting focused attention without unnecessary complexity.	1. Consistent 2D visual style.<br>2. Background music when appropriate.<br>3. Limited on-screen clutter.<br>4. Repetitive but goal-oriented interaction.<br>5. Dynamic elements in hybrid games while retaining simple task rules.
Social interaction (partially addressed)	Supporting interpersonal interaction where applicable.	1. Primarily single-player design.<br>2. Social interaction mainly occurs through therapist–patient supervision.<br>3. Multiplayer or remote competitive modes were not implemented.

B. GameFlow

The GameFlow model proposed by Sweetser and Wyeth [53] describes eight dimensions of player enjoyment: concentration, challenge, player skills, control, clear goals, feedback, immersion, and social interaction. Table III summarizes how these criteria were mapped to the corresponding design decisions.

User-centered principles were also applied during physical development, as observations from preliminary use directly informed changes in ergonomics, stability, and device configuration [19].

References

[1] A. L. van Ommeren, L. C. Smulders, G. B. Prange-Lasonder, J. H. Buurke, P. H. Veltink, and J. S. Rietman, "Assistive technology for the upper extremities after stroke: Systematic review of users' needs," *JMIR Rehabilitation and Assistive Technologies*, vol. 5, no. 2, 2018. [Online]. Available: https://www.sciencedirect.com/science/article/pii/S2369252918000200

[2] B. Sheng, J. Zhao, Y. Zhang, S. Xie, and J. Tao, "Commercial device-based hand rehabilitation systems for stroke patients: State of the art and future prospects," *Heliyon*, vol. 9, no. 3, p. e13588, 2023.

[3] A. Demofonti, G. Carpino, L. Zollo, and M. J. Johnson, "Affordable robotics for upper limb stroke rehabilitation in developing countries: A systematic review," *IEEE Transactions on Medical Robotics and Bionics*, vol. 3, no. 1, pp. 11–20, 2021.

[4] S. Hussain, P. K. Jamwal, P. Van Vliet, and M. H. Ghayesh, "State-of-the-art robotic devices for wrist rehabilitation: Design and control aspects," *IEEE Transactions on Human-Machine Systems*, vol. 50, no. 5, pp. 361–372, 2020.

[5] S. F. M. Toh, K. N. K. Fong, P. C. Gonzalez, and Y. M. Tang, "Application of home-based wearable technologies in physical rehabilitation for stroke: A scoping review," *IEEE Transactions on Neural Systems and Rehabilitation Engineering*, vol. 31, pp. 1614–1623, 2023.

[6] Y. Hutabarat, D. Owaki, and M. Hayashibe, "Recent advances in quantitative gait analysis using wearable sensors: A review," *IEEE Sensors Journal*, vol. 21, no. 23, pp. 26 470–26 487, 2021.

[7] A. Favata, R. Gallart-Agut, R. Pàmies-Vilà, C. Torras, and J. M. Font-Llagunes, "Imu-based systems for upper-limb kinematic analysis in clinical applications: A systematic review," *IEEE Sensors Journal*, vol. 24, no. 18, pp. 28 576–28 594, 2024.

[8] N. Gupta, V. Kasula, P. Sanmugananthan, N. Panico, A. H. Dubin, D. A. Sykes, and R. S. D'Amico, "Smartwear body sensors for neurological and neurosurgical patients: A review of current and future technologies," *World Neurosurgery: X*, vol. 21, p. 100247, 2024. [Online]. Available: https://www.sciencedirect.com/science/article/pii/S2590139723000960

[9] M. W. O'Dell, "Stroke rehabilitation and motor recovery," *Continuum (Minneapolis, Minn.)*, vol. 29, no. 2, pp. 605–627, 2023.

[10] D. Olcay and S. Ulusam Seckiner, "Gamification of upper limb tangible-wearable rehabilitation devices," in *Industrial Engineering in the Digital Disruption Era*, ser. Lecture Notes in Management and Industrial Engineering, F. Calisir and O. Korhan, Eds. Cham: Springer, 2020.

[11] J. Tosto-Macunso, L. Tabacof, J. Herrera, E. Breyman, S. Dewil, M. Cortes, L. Correa-esnard, C. Kellner, N. Dangayach, and D. Putrino, "Gamified neurorehabilitation strategies for post-stroke motor recovery: Challenges and advantages," *Current Neurology and Neuroscience Reports*, vol. 22, 03 2022.

[12] K. Thomson, A. Todhunter-Brown, C. Bugge, and M. Brady, "Commercial gaming devices for stroke upper limb rehabilitation: The stroke survivor experience," *Journal of Rehabilitation and Assistive Technologies Engineering*, vol. 7, p. 205566832091538, 05 2020.

[13] M. Babaiasl, S. Mahdioun, P. Jaryani, and M. Yazdani, "A review of technological and clinical aspects of robot-aided rehabilitation of upper-extremity after stroke," *Disability and Rehabilitation: Assistive Technology*, vol. 11, no. 4, pp. 263–280, 2016.

[14] L. Li, Q. Fu, S. Tyson, N. Preston, and A. Weightman, "A scoping review of design requirements for a home-based upper limb rehabilitation robot for stroke," *Topics in Stroke Rehabilitation*, vol. 29, no. 6, pp. 449–463, 2022.

[15] M. F. Shah, S. Hussain, R. Goecke, and P. K. Jamwal, "Mechanism design and control of shoulder rehabilitation robots: A review," *IEEE Transactions on Medical Robotics and Bionics*, vol. 5, no. 4, pp. 780–792, 2023.

[16] P. Tamayo-Serrano, S. Garbaya, and P. Blazevic, "Gamified in-home rehabilitation for stroke survivors: Analytical review," *International Journal of Serious Games*, vol. 5, 03 2018.

[17] F. S. Mohd Zuki, S. Sulaiman, D. R. A. Rambli, F. Merienne, and M. N. Mohamad Saad, "Sensory feedback and interactivity: Enhancing motivation and engagement for vr stroke rehabilitation," in *2021 International Conference on Computer '&' Information Sciences (ICCOINS)*, 2021, pp. 333–338.

[18] D. Charles, D. Holmes, T. Charles, and S. McDonough, "Virtual reality design for stroke rehabilitation," in *Biomedical Visualisation*, ser. Advances in Experimental Medicine and Biology, P. Rea, Ed., vol. 1235. Cham: Springer, 2020.

[19] J. Ambros-Antemate, M. del Pilar Beristain-Colorado, M. Vargas-Treviño, J. Gutiérrez-Gutiérrez, P. A. Hernández-Cruz, I. Gallegos-Velasco, and A. Moreno-Rodríguez, "Improving adherence to physical therapy in the development of serious games: Conceptual framework design study," *JMIR Formative Research*, vol. 7, p. e39838, 2023.

[20] J. J. Sánchez-Gil, A. Sáez-Manzano, R. López-Luque, J.-J. Ochoa-Sepúlveda, and E. Cañete-Carmona, "Gamified devices for stroke rehabilitation: A systematic review," *Computer Methods and Programs in Biomedicine*, p. 108476, 2024. [Online]. Available: https://www.sciencedirect.com/science/article/pii/S0169260724004693

[21] C. Ganter-Argast, M. Schipper, M. Shamsrizi, C. Stein, and R. Khalil, "The light side of gaming: creativity and brain plasticity," *Frontiers in Human Neuroscience*, vol. 17, p. 1280989, Jan 05 2024.

[22] F. Pereira, S. Bermúdez i Badia, C. Jorge *et al.*, "The use of game modes to promote engagement and social involvement in multi-user serious games: a within-person randomized trial with stroke survivors," *Journal of NeuroEngineering and Rehabilitation*, vol. 18, p. 62, 2021.

[23] B. Morschheuser, L. Hassan, K. Werder, and

J. Hamari, “How to design gamification? a method for engineering gamified software,” *Information and Software Technology*, vol. 95, pp. 219–237, 2018. [Online]. Available: https://www.sciencedirect.com/science/article/pii/S095058491730349X

[24] S. K. D, S. G. A, R. A. Raja, S. A. A, N. Nayak, and A. Arumugam, “Effects of a novel game-based arm rehabilitation device, the armable, on improving upper limb function in people with stroke,” in *2021 14th International Conference on Developments in eSystems Engineering (DeSE)*, 2021, pp. 259–264.

[25] M. Figeys, F. Koubasi, D. Hwang, A. Hunder, A. Miguel-Cruz, and A. Ríos Rincón, “Challenges and promises of mixed-reality interventions in acquired brain injury rehabilitation: A scoping review,” *International Journal of Medical Informatics*, vol. 179, p. 105235, 2023. [Online]. Available: https://www.sciencedirect.com/science/article/pii/S1386505623002538

[26] I. Laffont, J. Froger, C. Jourdan, K. Bakhti, L. E. van Dokkum, A. Gouaich, H. Y. Bonnin, P. Armingaud, A. Jaussent, M. C. Picot, E. Le Bars, A. Dupeyron, C. Arquizan, A. Gelis, and D. Mottet, “Rehabilitation of the upper arm early after stroke: Video games versus conventional rehabilitation. a randomized controlled trial,” *Annals of Physical and Rehabilitation Medicine*, vol. 63, no. 3, pp. 173–180, 2020. [Online]. Available: https://www.sciencedirect.com/science/article/pii/S1877065719301885

[27] N. Hocine, A. Gouaïch, S. A. Cerri *et al.*, “Adaptation in serious games for upper-limb rehabilitation: an approach to improve training outcomes,” *User Modeling and User-Adapted Interaction*, vol. 25, pp. 65–98, 2015.

[28] P. Wang, R. Koh, C. Boucharenc, and C. Yen, “Lights out: An interactive tangible game for training of post-stroke reaching,” 05 2016, pp. 1937–1944.

[29] S. Peng and Y. Guo, “Research on the intelligent interaction design of gamified hand rehabilitation training for the elderly with stroke,” in *Human Aspects of IT for the Aged Population*, Q. Gao and J. Zhou, Eds. Cham: Springer Nature Switzerland, 2025, pp. 335–348.

[30] S.-Y. Chien, C.-Y. Wu, A. M.-K. Wong, C.-K. Chen, and S. L. Beckman, “Exploring the impact of an interactive electronic pegboard on manual dexterity and cognitive skills of patients with stroke: Preliminary analysis,” *JMIR Form Res*, vol. 8, p. e55481, Oct 2024. [Online]. Available: https://formative.jmir.org/2024/1/e55481

[31] P. A. Rego, P. M. Moreira, and L. P. Reis, “Proposal of an extended taxonomy of serious games for health rehabilitation,” *GAMES FOR HEALTH JOURNAL: Research, Development, and Clinical Applications*, vol. 7, no. 5, 2018.

[32] V. Wattanasoontorn, I. Boada, R. García Hernandez, and M. Sbert, “Serious games for health,” *Entertainment Computing*, vol. 4, p. 231–247, 12 2013.

[33] P. Rego, P. M. Moreira, and L. P. Reis, “Serious games for rehabilitation: A survey and a classification towards a taxonomy,” in *5th Iberian Conference on Information Systems and Technologies*, 2010, pp. 1–6.

[34] S. Verschueren, C. Buffel, and G. Vander Stichele, “Developing theory-driven, evidence-based serious games for health: Framework based on research community insights,” *JMIR Serious Games*, vol. 7, no. 2, p. e11565, 2019.

[35] Z. Yang, S. Jie, L. Shiqi, C. Ping, and N. Shengjia, “Tangible interactive upper limb training device,” 05 2018, pp. 1–5.

[36] O. Mubin, F. Alnajjar, A. Mahmud, N. Jishtu, and B. Alsinglawi, “Exploring serious games for stroke rehabilitation: a scoping review,” *Disability and Rehabilitation: Assistive Technology*, vol. 17, pp. 1–7, 06 2020.

[37] C. Vieira, C. Ferreira da Silva Pais-Vieira, J. Novais, and A. Perrotta, “Serious game design and clinical improvement in physical rehabilitation: Systematic review,” *JMIR Serious Games*, vol. 9, no. 3, p. e20066, Sep 2021. [Online]. Available: https://games.jmir.org/2021/3/e20066

[38] S. Grudpan *et al.*, “Virtual reality games for stroke rehabilitation: A feasibility study,” in *Entertainment Computing – ICEC 2020*, ser. Lecture Notes in Computer Science, N. Nunes, L. Ma, M. Wang, N. Correia, and Z. Pan, Eds., vol. 12523. Cham: Springer, 2020.

[39] M. G. Arouca, I. B. D. C. Neves, M. E. Barreto, C. D. S. Cruz, and R. L. Brito, “Gamification frameworks and models for health contexts: an integrative review,” *International Journal for Innovation Education and Research*, vol. 9, no. 9, pp. 374–386, 2021.

[40] R. Baranyi, “Deapsea: Workflow-supported serious game design for stroke rehabilitation,” *International Journal of Computer Games Technology*, vol. 2023, pp. 1–15, 05 2023.

[41] N. M. Tuah, F. Ahmedy, A. Gani, and L. N. Yong, “A survey on gamification for health rehabilitation care: Applications, opportunities, and open challenges,” *Information*, vol. 12, no. 2, 2021. [Online]. Available: https://www.mdpi.com/2078-2489/12/2/91

[42] I. Dykens, A. Wetzel, S. Dorton, and E. Batchelor, “Towards a unified model of gamification and motivation,” in *Adaptive Instructional Systems. Design and Evaluation. HCII 2021*, ser. Lecture Notes in Computer Science, R. Sottilare and J. Schwarz, Eds., vol. 12792. Cham: Springer, 2021.

[43] S. M. Schöbel, A. Janson, and M. Söllner, “Capturing the complexity of gamification elements: a holistic approach for analysing existing and deriving novel gamification designs,” *European Journal of Information Systems*, vol. 29, no. 6, pp. 641–668, 2020.

[44] F. S. M. Zuki, F. Merienne, S. Sulaiman, A. Ricca, D. R. A. Rambli, and M. N. M. Saad, “Gamification, sensory feedback, adaptive function on virtual reality rehabilitation: a brief review,” in *2022 International Conference on Digital Transformation and Intelligence (ICDI)*, 2022, pp. 330–335.

[45] X. Huang, X. Xiang, Y. Liu, Z. Wang, Z. Jiang, and L. Huang, “The use of gamification in the self-management of patients with chronic diseases: Scoping review,” *JMIR Serious Games*, vol. 11, p. e39019, 2023.

[46] D. Johnson, S. Deterding, K.-A. Kuhn, A. Staneva, S. Stoyanov, and L. Hides, "Gamification for health and wellbeing: A systematic review of the literature," *Internet Interventions*, vol. 6, pp. 89–106, 2016. [Online]. Available: https://www.sciencedirect.com/science/article/pii/S2214782916300380

[47] K. Seaborn and D. I. Fels, "Gamification in theory and action: A survey," *International Journal of Human-Computer Studies*, vol. 74, pp. 14–31, 2015. [Online]. Available: https://www.sciencedirect.com/science/article/pii/S1071581914001256

[48] M. N. Dahdah, M. Bennett, P. Prajapati, T. D. Parsons, E. Sullivan, and S. Driver, "Application of virtual environments in a multi-disciplinary day neurorehabilitation program to improve executive functioning using the stroop task," *NeuroRehabilitation*, vol. 41, no. 4, pp. 721–734, 2017, pMID: 29254114.

[49] G. Richter, D. Raban, and S. Rafaeli, "Studying gamification: The effect of rewards and incentives on motivation," in *Gamification in Education and Business*, T. Reiners and L. Wood, Eds. Cham: Springer, 2015.

[50] K. Loughrey and D. O Broin, "Are we having fun yet? misapplying motivation to gamification," in *2018 IEEE Games, Entertainment, Media Conference (GEM)*, 2018, pp. 1–9.

[51] N. Barrett, I. Swain, C. Gatzidis, and C. Mecheraoui, "The use and effect of video game design theory in the creation of game-based systems for upper limb stroke rehabilitation," *Journal of Rehabilitation and Assistive Technologies Engineering*, vol. 3, p. 2055668316643644, 2016.

[52] R. M. Ryan and E. L. Deci, "Self-Determination Theory and the facilitation of intrinsic motivation, social development, and well-being," *American Psychologist*, vol. 55, no. 1, pp. 68–78, 2000.

[53] P. Sweetser and P. Wyeth, "Gameflow: a model for evaluating player enjoyment in games," *Comput. Entertain.*, vol. 3, no. 3, p. 3, jul 2005. [Online]. Available: https://doi.org/10.1145/1077246.1077253

[54] R. Cuthbert, S. Turkay, and R. Brown, "The effects of customisation on player experiences and motivation in a virtual reality game," in *OZCHI'19: 31ST AUSTRALIAN CONFERENCE ON HUMAN-COMPUTER-INTERACTION*, 12 2019, pp. 221–232.

[55] M. Böckle, J. Novak, and M. Bick, "Towards adaptive gamification: A synthesis of current developments," in *Proceedings of the 25th European Conference on Information Systems (ECIS)*, 2017. [Online]. Available: https://aisel.aisnet.org/ecis2017_rp/11/

[56] B. Botte, S. Bakkes, and R. Veltkamp, "Motivation in gamification: Constructing a correlation between gamification achievements and self-determination theory," in *Games and Learning Alliance*, I. Marfisi-Schottman, F. Bellotti, L. Hamon, and R. Klemke, Eds., vol. 12517. Cham: Springer International Publishing, 2020, pp. 157–166.
