# Supplementary material for: A Therapist-Configurable Serious Game System for Upper-Limb Neurorehabilitation: A Pilot Feasibility Study
Source: Sensors (Basel). 2026 Sep 20;26(18):5956. doi: 10.3390/s26185956 (PMC13611459; doi:10.3390/s26185956)
Supplement: Supplementary file 1 [file sensors-26-05956-s001.zip › Supplementary Material S2 - Kinematics.pdf]

Supplementary Material S2: Detailed Exploratory Movement Protocol, Session-Level Summaries, and Individualized Bilateral Adaptation

This supplementary material provides the complete methods and session-level descriptive results of the exploratory target-to-target movement analysis. It includes the rationale for participant selection, the standardized task sequence, condition order, video-based measurement procedure, data-aggregation method, and individualized bilateral adaptation used for IN_{10}. These analyses were hypothesis-generating and were not used to determine feasibility or clinical efficacy.

I. Participant selection and purpose

A standardized target-to-target comparison was conducted in IN_6, IN_8, and IN_{11}. These participants had unilateral upper-limb impairment and the lowest baseline BBT scores among those for whom the standardized protocol could be applied. The subgroup was selected purposively to explore movement performance in participants with marked initial manual-dexterity limitations and was not intended to represent the complete clinical cohort.

IN_{10} was not included in the quantitative comparison because bilateral upper-limb impairment required an individualized protocol. This adaptation is described separately below.

II. Standardized protocol

Data were collected during Sessions 3–6, after participants had completed the initial familiarization sessions. The exploratory protocol occupied approximately the first 5–6 min of each scheduled session, and the time assigned to the remaining game activities was adjusted so that the assessment did not extend the planned session duration.

Two conditions were evaluated: a non-gamified instruction condition and a gamified condition. Both used the same target positions and color sequence. At the beginning of each condition, the handle was placed on the blue target, and a valid blue-target detection established the initial event without being counted as a movement. Participants then completed three cycles comprising blue-to-red, red-to-pink, and pink-to-blue movements, producing nine target-to-target segments per condition.

In the instruction condition, the therapist or researcher verbally indicated the target sequence. In the game condition, a hybrid serious game required the same sequence and number of movements. Game difficulty was set to Easy in Session 3, Intermediate in Session 4, and Hard in Sessions 5 and 6.

Condition order was alternated across sessions to partially reduce systematic order-related fatigue or learning effects. The instruction condition was performed first in Sessions 3 and 5, whereas the game condition was performed first in Sessions 4 and 6.

III. Video-based measurement

Movement time was defined as the elapsed time between two consecutive valid RGB target-detection events recorded by the system. Concurrently, sessions were recorded using an external camera maintained in a fixed position. A ruler was fixed within the recorded workspace, and a white marker placed at the distal tip of the handle served as the visual reference.

The recordings were reviewed manually. For each segment, the starting and ending positions of the marker were read against the ruler. The absolute difference between these positions was recorded in centimetres as the video-estimated one-dimensional projected displacement. Derived mean speed was calculated separately for each segment by dividing its projected displacement by its movement time.

No automated tracking, formal camera calibration, perspective correction, continuous trajectory reconstruction, or two- or three-dimensional kinematic analysis was performed. The measurements do not represent total path length, joint range of motion, instantaneous speed, trajectory efficiency, or movement smoothness. Potential error due to perspective, parallax, marker interpretation, or manual ruler reading cannot be excluded. All 216 planned target-to-target segments were measurable and included in the descriptive summaries.

IV. Data aggregation

Each participant completed nine movement segments per condition and session. Session-level values were calculated by averaging the nine segment-level observations. Participant-level summaries combining Sessions 3–6 were therefore based on 36 segments per condition and participant.

Derived mean speed was first calculated separately for each segment and subsequently averaged. Consequently, the reported mean speed is not necessarily equal to the reported mean displacement divided by the reported mean movement time. No inferential comparison between conditions was conducted because participants were selected purposively and contributed repeated observations.

V. Participant-level descriptive summaries

Figure 1 presents the participant-level mean values obtained by combining Sessions 3–6, corresponding to 36 movement segments per condition and participant. Table I shows all data.

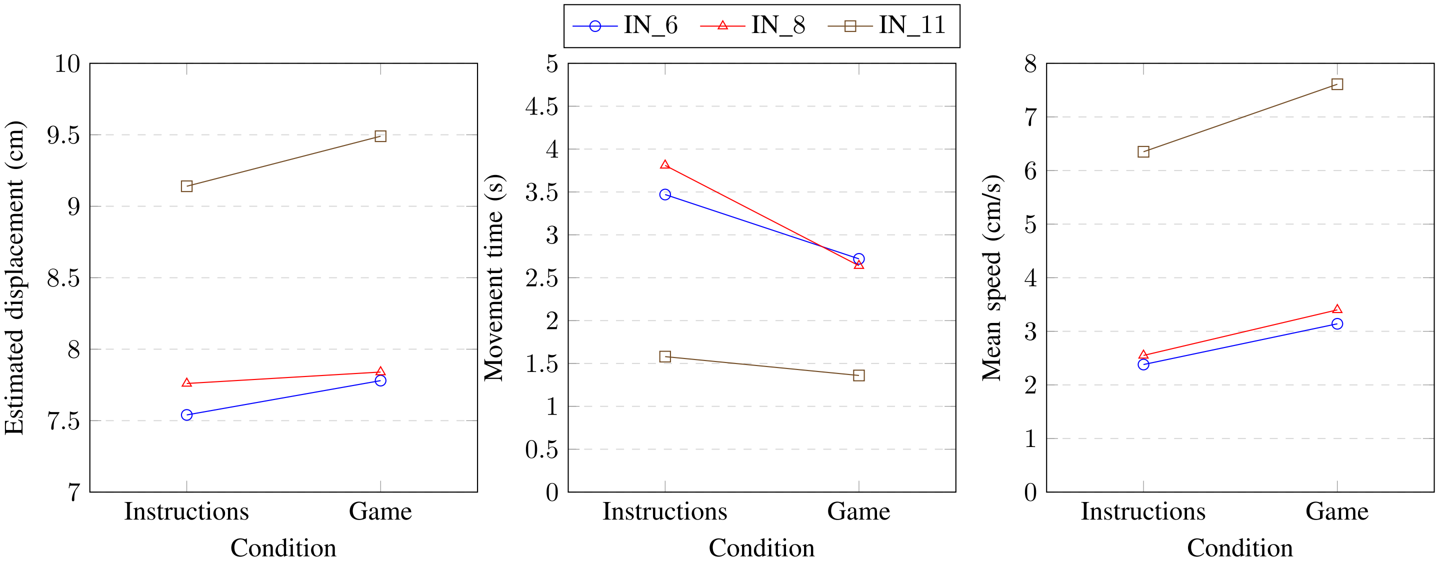

Fig. 1: Global comparison between the instructions and game conditions for each participant. Each panel shows video-estimated movement displacement, movement time, and derived mean speed, with Sessions 3–6 combined.

TABLE I: Session-wise summary of segment-level movement variables for each participant, task condition, and game difficulty. Data correspond to intervention sessions 3–6. Values represent the mean of the nine target-to-target movements completed in each condition and session (n = 9).

Patient	Session	Condition	Difficulty	Estimated displacement (cm)	Movement time (s)	Derived mean speed (cm/s)
IN_6	S3	Instructions	–	7.18	3.56	2.47
	S3	Game	Easy	7.78	3.00	2.59
	S4	Game	Intermediate	8.13	3.11	2.68
	S4	Instructions	–	8.13	3.22	2.55
	S5	Instructions	–	7.18	3.89	2.08
	S5	Game	Hard	7.74	1.89	4.35
	S6	Game	Hard	7.46	2.89	2.93
	S6	Instructions	–	7.68	3.22	2.43
IN_8	S3	Instructions	–	7.49	5.44	1.77
	S3	Game	Easy	7.57	3.11	2.52
	S4	Game	Intermediate	8.19	3.56	2.35
	S4	Instructions	–	7.52	3.89	2.06
	S5	Instructions	–	8.06	3.56	2.41
	S5	Game	Hard	7.92	2.00	4.17
	S6	Game	Hard	7.69	1.89	4.55
	S6	Instructions	–	7.98	2.33	3.96
IN_{11}	S3	Instructions	–	9.51	1.78	6.30
	S3	Game	Easy	9.03	1.56	6.36
	S4	Game	Intermediate	9.64	1.56	6.69
	S4	Instructions	–	9.29	2.00	4.82
	S5	Instructions	–	9.44	1.44	6.71
	S5	Game	Hard	8.94	1.11	8.52
	S6	Game	Hard	10.32	1.22	8.89
	S6	Instructions	–	8.32	1.11	7.59

VI. Exploratory bilateral case adaptation

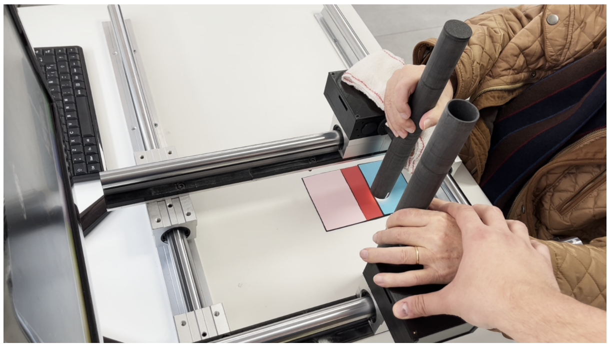

(a) Position 1

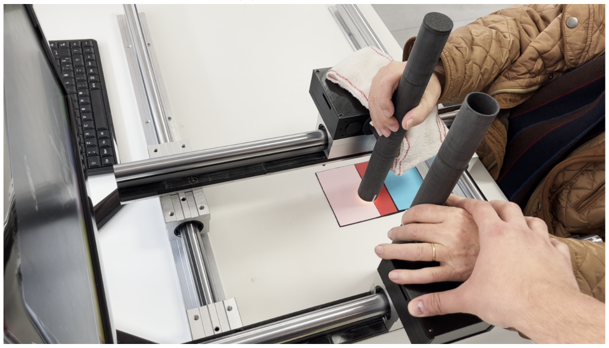

(b) Position 2

Fig. 2: Uncoupled bilateral configuration used by participant IN_{10} after removal of the mechanical connector. During the illustrated gamified task, the right, more-affected arm controlled the game, and target spacing was individualized according to the participant's available movement range.

Participant IN_{10} had bilateral upper-limb impairment, with greater impairment of the right arm. During Sessions 1 and 2, the two handles were mechanically coupled so that movement generated by the less-affected arm assisted the more-affected arm. From Session 3 onward, the connector was removed and the handles were used independently. Mechanical coupling therefore did not form part of the Session 3–6 exploratory protocol described above, and IN_{10} was not included in the standardized quantitative comparison.

During gamified practice, the right, more-affected arm controlled the game, and target positions were individualized according to the participant's available movement range. Figure 2 shows the uncoupled configuration. Supplementary Video 2 includes recordings illustrating the individualized bilateral protocol (also available at: https://youtu.be/ffFRkTsI6t8.).

After removal of the connector, voluntary movement of the more-affected arm remained limited during instruction-based practice, whereas broader and more goal-directed movements were observed descriptively during gamified practice. Because this was a single individualized case without a standardized quantitative comparison, this observation is reported solely as contextual, hypothesis-generating information.
