# Supplementary material for: A Therapist-Configurable Serious Game System for Upper-Limb Neurorehabilitation: A Pilot Feasibility Study
Source: Sensors (Basel). 2026 Sep 20;26(18):5956. doi: 10.3390/s26185956 (PMC13611459; doi:10.3390/s26185956)
Supplement: Supplementary file 1 [file sensors-26-05956-s001.zip › Supplementary Material S3 - Support Cohorts.pdf]

Supplementary Material S3: Preliminary Support-Sample Procedures and Descriptive Results

I. Overview

Before the clinical feasibility study, two preliminary support samples were used during system refinement under progressively more realistic conditions. The sample historically designated Cohort C comprised healthy young adults and was used primarily for technical robustness testing. The sample designated Cohort B comprised older nursing-home residents and was used to identify age-related usability and implementation barriers before clinical deployment.

These samples were not part of the clinical feasibility analysis, were not designed for hypothesis testing, and cannot be formally compared with the neurological cohort reported in the main manuscript. Their procedures and descriptive findings are retained in this supplementary material exclusively to document the system-refinement process transparently.

II. Support-Cohort Procedures

A. Cohort C: Healthy Young Adults

Cohort C consisted of 25 healthy young adults, all men, with a mean age of 24.52 years (SD = 5.75). Each participant completed a single supervised session of approximately 30 minutes.

The initial minutes were devoted to informed consent and standardized instructions. Participants subsequently interacted with up to five serious games. Throughout each session, the researcher maintained a structured technical incident log documenting software failures, unexpected game behavior, perceived interaction delays, system resets, and mechanical or interaction-related issues.

No usability or game-experience questionnaires were administered in this cohort because its primary purpose was technical robustness testing before involving older and neurological participants.

B. Cohort B: Nursing Home Residents

Cohort B consisted of seven older adults residing in a nursing home. Participants completed four supervised sessions of approximately 30 minutes each, scheduled twice per week over two weeks. Figure S1 shows elderly residents of the nursing home interacting with the device during a gaming session.

This support stage was designed to evaluate repeated system use in an older population and to identify usability barriers potentially associated with age, frailty, visual limitations, task comprehension, or interaction speed. Selected game parameters, including reaction times, visual presentation, and comprehension demands, were refined when necessary on the basis of observations collected during these sessions.

Perceived usability was assessed using the System Usability Scale (SUS) after completion of the four-session program. Motivation was assessed using the 9-item Intrinsic Motivation Inventory (IMI) after Sessions 2 and 4. Session logs were also used to document assistance, pauses, fatigue, acceptance, and technical incidents.

Following completion of participant testing, four members of the nursing-home care staff participated in a brief semistructured interview addressing ease of use, potential implementation in routine care, perceived usefulness, and practical barriers. Responses were summarized descriptively without formal qualitative coding or thematic analysis.

III. Cohort B Participant Characteristics

Participant characteristics are reported in Table S1. Three residents had a history of chronic ischemic stroke, one had hand osteoarthritis, and the remaining participants had no relevant upper-limb pathology reported for the purposes of this study.

Participants ranged in age from 76 to 95 years, with a mean age of 87.0 years (SD = 7.1). The residents with chronic stroke were retained within Cohort B because this cohort was defined according to the residential usability setting rather than as a clinical stroke cohort.

IV. Support-Cohort Results

A. Cohort C: Technical Robustness

A total of 25 preliminary sessions were completed in Cohort C, corresponding to approximately 12.5 h of scheduled session time.

During this stage, 27 technical and game-related optimization points were identified and subsequently corrected. Most were associated with game logic and software behavior, including incorrect object behavior, unintended sprite deletion during movement, inconsistencies between participant performance and the reward displayed, and errors in visual feedback following correct or incorrect responses.

The identified issues were corrected during this preliminary stage before the system was introduced into the older-adult support cohort.

B. Cohort B: Technical and Operational Findings

A total of 28 sessions were completed in the nursing-home cohort, corresponding to approximately 14 h of scheduled session time.

Three additional technical/usability issues were recorded during this stage. All were related to the small visual size of selected sprites or graphical elements in some serious games. These observations led to adjustments in the presentation of the corresponding game elements.

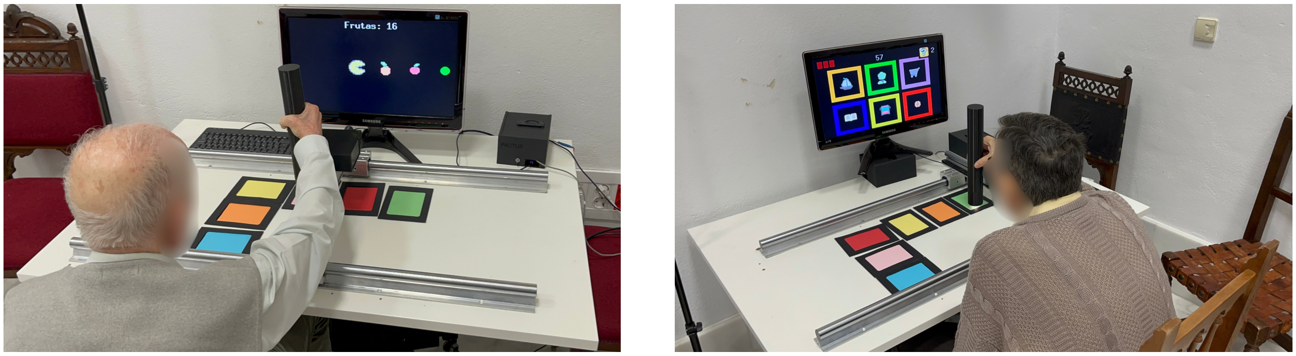

(a) User 1 (b) User 2

Fig. S1: Two older adults interacting with the device.

TABLE S1: Characteristics of the nursing-home support cohort (Cohort B).

	**Age (years)**	**Sex**	**Relevant condition**
NH_1	83	Female	Chronic ischemic stroke (1 year)
NH_2	83	Male	Chronic ischemic stroke (3 years)
NH_3	93	Male	Chronic ischemic stroke (2 years)
NH_4	76	Male	None reported
NH_5	85	Female	Hand osteoarthritis
NH_6	95	Female	None reported
NH_7	94	Female	None reported

C. *Cohort B: Perceived Usability*

The seven nursing-home participants obtained a mean SUS score of 69.6 points (SD = 11.1). Individual scores ranged from 57.5 to 85.0 and are shown in Figure S2.

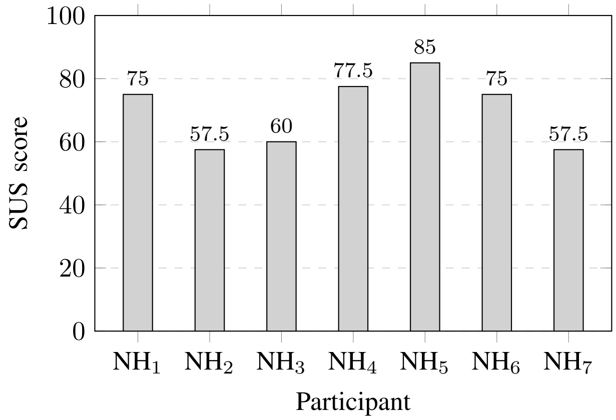

Fig. S2: Individual System Usability Scale (SUS) scores in the nursing-home support cohort (n = 7).

D. *Cohort B: Motivation*

IMI subscale scores obtained after Sessions 2 and 4 are summarized in Table S2. Interest/enjoyment scores were 6.14 (SD = 0.72) at the first assessment and 6.29 (SD = 0.60) at the second assessment. Perceived competence scores were 6.00 (SD = 0.76) and 5.86 (SD = 0.48), respectively. Pressure/tension scores were 2.14 (SD = 1.25) and 2.07 (SD = 0.93).

TABLE S2: IMI subscale scores for the nursing-home support cohort (n = 7), assessed after Sessions 2 and 4.

**Subscale**	**Assessment**	**Mean**	**SD**	**Range**
Interest/enjoyment	Session 2	6.14	0.72	4.8–6.8
	Session 4	6.29	0.60	5.0–6.8
Perceived competence	Session 2	6.00	0.76	5.0–7.0
	Session 4	5.86	0.48	5.0–6.5
Pressure/tension	Session 2	2.14	1.25	1.0–4.0
	Session 4	2.07	0.93	1.0–3.5

E. *Nursing-Home Staff Feedback*

Feedback was obtained from four members of the nursing-home care staff: two nursing assistants, one geriatric care assistant, and one nurse. Three respondents were women and one was a man.

All four staff members reported positive perceived value of the system, with particular emphasis on its potential use for cognitive stimulation in older adults. They also considered the interaction relatively easy to learn and described the system

as a potential tool for repeated use if it were available in the facility.

The principal implementation barriers mentioned during the interviews concerned visual accessibility and physical space. Two staff members referred to the size of some sprites and to screen visibility as potential limitations for residents with reduced vision. Two also mentioned the amount of table space required by the system, although they indicated that an appropriate area was available within the facility.

Table S3 summarizes the main topics identified from these interviews.

V. Summary

The preliminary support cohorts were used to refine the system before implementation in the primary clinical population. Cohort C identified 27 technical and game-related optimization points during the initial robustness stage. Following these corrections, only three additional usability issues were identified during repeated use in Cohort B, all related to the visual size of selected game elements.

The nursing-home cohort obtained a mean SUS score of 69.6 (SD = 11.1), while the descriptive IMI results remained relatively stable between Sessions 2 and 4. Staff feedback additionally identified visual accessibility and physical space as the principal implementation considerations in the residential setting.

These findings are reported as supporting technical and usability information and were not used for formal comparison with the neurological clinical cohort evaluated in the main manuscript.

TABLE S3: Summary of feedback from nursing-home care staff (n = 4).

Topic	Summary of feedback	Frequency when explicitly reported
Perceived usefulness	Staff considered the system potentially useful as a complementary activity, particularly for cognitive stimulation and repeated practice in older adults.	4/4 positive overall
Ease of learning	The system was generally described as straightforward to learn and potentially suitable for repeated use in the nursing-home environment.	–
Visual accessibility	Some sprites and on-screen elements were considered too small for residents with reduced vision.	2/4
Space requirements	The physical footprint of the tabletop setup was identified as a practical consideration, although suitable space was reported to be available.	2/4
